# Coding and Non-coding RNAs: Molecular Basis of Forest-Insect Outbreaks

**DOI:** 10.3389/fcell.2020.00369

**Published:** 2020-06-11

**Authors:** Sufang Zhang, Sifan Shen, Zhongwu Yang, Xiangbo Kong, Fu Liu, Zhang Zhen

**Affiliations:** ^1^Key Laboratory of Forest Protection of State Forestry and Grassland Administration, Research Institute of Forest Ecology, Environment and Protection, Chinese Academy of Forestry, Beijing, China; ^2^Forestry Comprehensive Development Center of Guilin, Guilin, China

**Keywords:** forest insect, outbreak, whole-transcriptome sequencing, lncRNA, ceRNA

## Abstract

Insect population dynamics are closely related to ‘human’ ecological and economic environments, and a central focus of research is outbreaks. However, the lack of molecular-based investigations restricts our understanding of the intrinsic mechanisms responsible for insect outbreaks. In this context, the moth *Dendrolimus punctatus* Walker can serve as an ideal model species for insect population dynamics research because it undergoes periodic outbreaks. Here, high-throughput whole-transcriptome sequencing was performed using *D. punctatus*, sampled during latent and outbreak periods, to systemically explore the molecular basis of insect outbreaks and to identify the involved non-coding RNA (ncRNA) regulators, namely microRNAs, long non-coding RNAs, and circular RNAs. Differentially expressed mRNAs of *D. punctatus* from different outbreak periods were involved in developmental, reproductive, immune, and chemosensory processes; results that were consistent with the physiological differences in *D. punctatus* during differing outbreak periods. Targets analysis of the non-coding RNAs indicated that long non-coding RNAs could be the primary ncRNA regulators of *D. punctatus* outbreaks, while circular RNAs mainly regulated synapses and cell junctions. The target genes of differentially expressed microRNAs mainly regulated the metabolic and reproductive pathways during the *D. punctatus* outbreaks. Developmental, multi-organismal, and reproductive processes, as well as biological adhesion, characterized the competing endogenous RNA network. Chemosensory and immune genes closely related to the outbreak of *D. punctatus* were further analyzed in detail: from their ncRNA regulators’ analysis, we deduce that both lncRNA and miRNA may play significant roles. This is the first report to examine the molecular basis of coding and non-coding RNAs’ roles in insect outbreaks. The results provide potential biomarkers for control targets in forest insect management, as well as fresh insights into underlying outbreak-related mechanisms, which could be used for improving insect control strategies in the future.

## Key Message

Understanding how an outbreak of insects happens is a key topic of forest pest research, but the underpinning molecular mechanisms remain unclear. We systemically analyzed the gene expression differences and their non-coding regulators, and deduced the corresponding developmental, reproductive, chemosensory, and immune processes as key terms related to pest outbreaks of a moth. This work deepens our understanding of outbreak-related mechanisms and supplies potential biomarkers or control targets for forest insect management.

## Introduction

As the most abundant and diverse animals on earth, insects play important roles in ecosystems, and their population dynamics are closely related to humans’ ecological and economic environments. Outbreaks are the central focus of insect population research, long attracting the attention of both entomologists and ecologists (Hunter, 1995). Investigations on why insect outbreaks occur have been performed, considering such reasons as nutrition (Quezada García et al., 2015), natural enemies (Berryman et al., 1987), environmental factors (Veran et al., 2015; Fuentealba et al., 2017), host resistance (Elderd et al., 2013), and population interactions (Lu et al., 2010). Yet, in the same habitat, only small numbers of species may undergo an outbreak, while most of the other co-occurring species maintain low and stable population sizes within the community (Alison, 1991). This indicates that outbreaking species may respond to changes in biological and/or abiotic factors differently from that of non-outbreaking species, and the former may have an intrinsic motivation differing from the latter (Myers, 1993). To date, the physiological responses of outbreak species to some factors have been heavily researched (Berryman, 1988; Veran et al., 2015); however, the molecular basis of these responses is still unclear, which limits our understanding of the intrinsic mechanisms responsible for insect outbreaks.

*Dendrolimus* spp. (Lepidoptera; Lasiocampidae) are major destructive pests of conifer forests (Xiao, 1992) and *Dendrolimus punctatus* Walker is the most widely distributed species in China (Chen, 1990; Billings, 1991; Luo et al., 2018), making it the most important pine tree defoliator. Notably, *D. punctatus* is a periodic outbreaking species; once it outbreaks, the caterpillars feeding on pine needles can quickly cause the large-scale destruction of pine forests, in a phenomenon called “Fire without smoke.” *D. punctatus* present different physiological characteristics during different outbreak stages. During the low-density latent period, the egg production and female ratio of *D. punctatus* are maintained at a certain level; during the ascending period, the egg production, female ratios, and population density increase considerably, with the degree of population aggregation also increasing; in the declining period, the body size, emergence rate, and female ratio of pine caterpillars all decrease to very low levels, while their rates of parasitism rise to a high level, additionally, their density drops rapidly to a very low level (Billings, 1991; Zhang et al., 2002). Collectively, these characteristics indicate that population density and several physiological characteristics, such as reproduction, immunity, or chemosensory system, are closely linked to the outbreak stages of *D. punctatus*. Outbreak of other insects also infer the importance of these physiological characteristics (Guo et al., 2011; Wang et al., 2013). Thus, *D. punctatus* offers a good model insect for investigating forest outbreak mechanisms, though the genetic mechanisms underlying this phenomenon remain generally unexplored.

With the advent of modern sequencing technology and acquisition of genomic information on outbreaking species, studies of insect outbreak mechanisms can now move from their ecological and physiological levels to understanding their basis at the molecular level (Kang et al., 2004; Wang et al., 2014). Accordingly, it is essential to investigate the molecular mechanisms that drive the occurrence of outbreak insects at multiple stages of an outbreak. Furthermore, biological processes are regulated not only by protein coding genes but also by non-coding RNA (ncRNA), such as microRNA (miRNA), long non-coding RNA (lncRNA), and circular RNA (circRNA) (Costa, 2005; Hansen et al., 2013; Beermann et al., 2016; Hombach and Kretz, 2016). The miRNAs—small ncRNAs of 20–30 nt—are well-known regulators of insect development and reproduction (Wei et al., 2009; He et al., 2016; Belles, 2017; Roy et al., 2018). LncRNAs are ncRNA longer than 200 bp (Amaral et al., 2008; Chen et al., 2016; Wu et al., 2016), which play important epigenetic regulating functions because they can form complexes with chromatin regulators at appropriate genomic regions in the cis and trans forms (Kretz et al., 2012; Mercer and Mattick, 2013). The circRNAs are newly discovered ncRNAs that form rings without the 5′-cap and 3′-polyadenylated tail (Wilusz and Sharp, 2013; Ling-Ling and Li, 2015), and they can originate from exons, introns, or intergenic regions (Zhang et al., 2013; Jeck and Sharpless, 2014; Li et al., 2015). Both lncRNAs and circRNAs can regulate gene expression through lncRNA/circRNA–miRNA–gene networks and they are also called “miRNA sponges” (Hansen et al., 2013). In particular, circRNA can regulate the splicing, transcription, and expression of genes, and subsequently regulate physiological characteristics (Li et al., 2015; Wang et al., 2017). With the development of next-generation sequencing technologies and bioinformatics, it is now possible to explore the molecular basis of dynamic population changes of outbreaking insects and to analyze the various regulators and modifiers of gene expression levels, such as miRNA, lncRNA, and circRNA, as well as the networks formed among different regulators and protein-coding genes (Yvonne et al., 2014; Li et al., 2017a; Wang et al., 2019).

In this comprehensive study, high-throughput whole-transcriptome sequencing was performed using populations of *D. punctatus* at low-density (latent period) and high-density (outbreak period) to systemically explore the molecular basis of different physiological characteristics during these two periods and to identify the involved regulating lncRNAs, circRNAs, and miRNAs. Furthermore, the chemosensory and immune genes and their regulating ncRNAs, which maybe closely linked to outbreak stages of *D. punctatus*, were analyzed in detail. This study is the first to not only identify the ncRNAs in *D. punctatus* but also explore the coding and non-coding RNA-based molecular mechanisms of forest insect outbreaks. These results will enhance our understanding of the unique ecological and evolutionary characteristics of the population dynamics-related mechanisms of outbreak insects.

## Materials and Methods

### Sampling, RNA Extraction, and RNA Quantification

*Dendrolimus punctatus* pupae of different densities during different outbreak stages (low-density latent period and high-density outbreak period) were collected from Quanzhou, Guilin City, in Guangxi Province, China. The low-density insects was collected from an area where less than 10% of the trees were damaged by *D. punctatus*, and the insects were rare on the damaged trees; the high-density insects was collected from an area where more than 80% of the trees were damaged by *D. punctatus*; many insects were visible on these pine tree and so they were easy to collect. These two sampling areas were located in two neighboring villages of the same town (low-density *D. punctatus*: ca. N25°58′4.72″, E111°14′47.19″; high-density *D. punctatus*: ca. N25°49′36.90″, E111°21′21.95″). All the sample specimens were collected from host Masson pine (*Pinus massoniana*) trees (ca. 10-years old) Approximately 50 pupae were collected from each low- or high-density population, and maintained in our laboratory under a 16-h light:8-h dark photoperiod at 26 ± 2°C and 50% ± 10% relative humidity. Newly emerged male and female *D. punctatus* were collected and immediately frozen in liquid nitrogen. Four groups were used: males of low-density, females of low-density, males of high-density, and females of high-density. Three biological replications were prepared for each sample.

Total RNA per sample was extracted using TRIzol (Invitrogen, Carlsbad, CA, United States). RNA degradation and contamination, especially with DNA, were checked using 1.5% agarose gels. The concentration and purity of RNA were both measured by a NanoDrop 2000 spectrophotometer (Thermo Fisher Scientific, Wilmington, DE, United States), and its integrity was assessed using the RNA Nano 6000 Assay Kit of the Agilent Bioanalyzer 2100 System (Agilent Technologies, CA, United States).

### Library Preparation for lncRNA-Seq, Clustering, and Sequencing

From each sample, a total of 1.5 μg RNA was used as input material after first removing the ribosomal RNA (rRNA) with the Ribo-Zero rRNA Removal Kit (Epicentre, Madison, WI, United States). Sequencing libraries were generated using the NEBNext^®^ Ultra^TM^ Directional RNA Library Prep Kit for Illumina^®^ (NEB, United States), by following the manufacturer’s recommendations. Unique index codes were added to attribute the sequences to each sample. Briefly, fragmentation was carried out using divalent cations under an elevated temperature in the NEBNext First Strand Synthesis Reaction Buffer (5×). First-strand cDNA was synthesized using a random hexamer primer and reverse transcriptase; next, second-strand cDNA synthesis was performed using DNA polymerase I and RNase H. The remaining overhangs were converted into blunt ends through exonuclease/polymerase activities. After the adenylation of the DNA fragments’ 3′ ends, NEB Next adaptors with hairpin-loop structures were ligated to them to prepare for the hybridization. To select insert fragments that were preferentially 150–200 bp in length, library fragments were first purified with AMPure XP beads (Beckman Coulter, Beverly, MA, United States). Then, 3 μL of USER enzyme (NEB) was used with the size-selected, adaptor-ligated cDNA, at 37°C for 15 min, before running the PCR. Then, the PCR was performed using Phusion High-Fidelity DNA polymerase, universal PCR primers, and an index (X) primer. Ensuing PCR products were then purified (AMPure XP system) and library quality assessed on an Agilent Bioanalyzer 2100.

The clustering of index-coded samples was done in an acBot Cluster Generation System, with a TruSeq PE Cluster Kitv3-cBot-HS (Illumina), according to the manufacturer’s instructions. After this cluster generation, the library preparations were sequenced on an Illumina HiSeq platform, and paired-end reads were generated.

### Library Preparation for Small RNA-Seq, Clustering, and Sequencing

For the small RNA library preparation, 1.5 μg of total RNA per sample was used with an NEBNext^®^ Multiplex Small RNA Library Prep Set for Illumina^®^ (NEB), by following the manufacturer’s recommendations, to which index codes were added to attribute the sequences to each sample.

Briefly, the 3′ SR and 5′ SR adaptors were ligated, and M-MuLV reverse transcriptase was used to synthesize the first stand chain. Then, PCR amplifications were performed using Long AmpTaq 2 × Master Mix, an SR primer, and an index (X) primer. PCR products were purified on 8% polyacrylamide gels (run at 100 V, for 80 min). DNA fragments of 140–160 bp (i.e., length of small ncRNA plus the 3′ and 5′ adaptors) were recovered and dissolved in an 8-μL elution buffer. Finally, all the PCR products were purified by the AMPure XP system, and library quality assessed.

The clustering of these index-coded samples was performed in a cBot Cluster Generation System, with a TruSeq PE Cluster Kit v4-cBot-HS (Illumina), according to the manufacturer’s instructions. After this cluster generation, each library preparation was sequenced on an Illumina HiSeq 2500 platform and 50-bp single-end reads generated.

### Quality Control

Raw data (i.e., raw reads) in the ‘fastq’ format were first processed using in-house perl scripts. In this step, clean data were obtained by removing from the raw data those reads containing adapters and poly-Ns, as well as any low quality reads. Additionally, the Q20, Q30, GC-content, and sequence duplication level of the clean data were calculated. For small RNA-Seq data, reads were trimmed and cleaned by removing sequences shorter than 15 nt or longer than 35 nt. Finally, at least 16.28 Gb of clean data for each lncRNA sequencing sample, with a Q30 > 92.99%, and more than 14.30 Mb of clean data for each small RNA-Seq sample, with a Q30 > 99.43%, were obtained. All downstream analyses were thus performed using only high-quality clean data.

### lncRNA Identification

The transcriptome was assembled using StringTie (v1.3.1) software^1^, based on the reads mapped to the reference genome of *D. punctatus* (Daehwan et al., 2015; Pertea et al., 2016). The assembled transcripts were then annotated using the gff compare program^2^. The unknown transcripts were used to screen for putative lncRNAs. To sort the ncRNA candidates among the unknown transcripts, four computational approaches, namely CPC/CNCI/Pfam/CPAT, were combined and used (Lei et al., 2007; Kai et al., 2013; Liang et al., 2013; Finn et al., 2014). Those transcripts exceeding 200 nt in length, and having more than two exons, were selected as lncRNA candidates (Kelley and Rinn, 2012; Lv et al., 2013); these were further screened using CPC/CNCI/Pfam/CPAT, which has the ability to distinguish not only protein-coding from non-coding genes but also different types of lncRNAs—including lincRNA, intronic lncRNA, anti-sense lncRNA, sense lncRNA.

### miRNA Identification

Use the Bowtie tools software (Langmead and Salzberg, 2012), clean reads were used as queries against the Silva, GtRNAdb, Rfam, and Repbase databases for sequence alignments, and to filter out rRNA, transfer RNA, small nuclear RNA, small nucleolar RNA, other ncRNAs as well as any repeats. The remaining reads were used to detect miRNAs through comparisons with *D. punctatus* genome (Zhang et al., 2020) and known miRNAs from the miRBase (v21)^3^, and novel miRNA were predicted by miRDeep3 (Friedländer et al., 2012).

### CircRNA Identification and Target Prediction

CircRNA Identifier (CIRI) was used to predict the circRNAs (Yang Y. et al., 2017). CIRI scans the SAM files, twice. In the first scan, CIRI detects junction reads of circRNA candidates, by using paired-end mapping and GT–AG splicing signals. Then, it re-scans the SAM alignment to eliminate false-positive candidates derived from incorrectly mapped reads of homologous genes or repetitive sequences. We used the miRanda (Doron et al., 2008) and RNA hybrid (Rehmsmeier et al., 2004) tools for predicting circRNA target miRNAs and the corresponding target genes of miRNAs.

### Expression Analysis

StringTie (v1.3.1) was used to calculate the fragments per kilo-base of exon per million fragments mapped (FPKM) values, for both lncRNAs and coding genes, in each sample (Pertea et al., 2016). Gene FPKMs were computed by summing the FPKMs of transcripts in each gene group; FPKMs were calculated based on the length of the fragments and the read counts mapped to this fragment. The expression levels of miRNA and circRNA were each quantified using TPM (transcript per million) (Fahlgren et al., 2007).

### Differential Expression Analysis

The differential expression analysis was performed using the ‘DESeq’ (v1.10.1) (Anders and Huber, 2012). DESeq provides statistical routines for determining differential expression in digital gene expression data, by using a model based on the negative binomial distribution. The resulting *P*-values are adjusted using the Benjamini and Hochberg’s approach (Haynes, 2013) for controlling the false discovery rate (FDR). Genes with an adjusted *P*-value < 0.01 and absolute value of log_2_ (fold-change) > 1 were designated as differentially expressed.

### Gene Functional Annotation

The mRNAs and targets of ncRNAs were queried in a BLAST algorithm-based search against the NR, SWISSPROT, KEGG, and KOG databases (using cut-off threshold of 1e-5), from which the most similar sequence targets were selected for functional annotations. Then, the molecular functions, biological processes, and cellular components of the genes were assigned a gene ontology (GO) annotation by Blast2GO (Stefan et al., 2008). The GO enrichment analysis of differentially expressed genes (DEGs) was carried out using the ‘topGO’ (Alexa and Rahnenfuhrer, 2010), and KOBAS software (Xie et al., 2011) was used to test for the statistical enrichment of these DEGs in KEGG pathways.

### Competing Endogenous RNA (ceRNA) Network Analysis

Differentially expressed ncRNAs and mRNAs between low- and high-density *D. punctatus* groups were also analyzed. The miRNA response elements were first identified, by screening the circRNA, lncRNA, and mRNA sequences. Then, the STRING database (Cytoscape 3.4.0) was used to identify protein–protein interactions among the products of these DEGs.

### Validation by Real Time Quantitative PCR (qRT-PCR)

To verify our RNA-Seq results, a total of thirteen DEGs, three lncRNAs, three miRNAs, and three circRNAs, were randomly selected to detect their respective expression levels by qRT-PCR. The RNAs used for this validation were the same as those of Illumina-sequenced RNAs. PimeScipt RT reagent Kit (TaKaRa, Dalian, China) was used to synthase cDNA (for mRNA, lncRNA and circRNA) with random hexamers. Mir-X^TM^ miRNA First Strand Synthesis and the SYBR^®^ qRT-PCR Kit (Takara, Dalian, China) were used to synthesize the DNA template for miRNA, for which miRNA primers were designed according to the Kit’s manual. The qRT-PCR was carried out with the SYBR Green PCR kit (TaKaRa, Dalian, China). Beta-actin (for mRNA, lncRNA, and circRNA) and U6 (for miRNA) served as controls. All primers used for qRT-PCR can be found in Supplementary Table S1. All qRT-PCR reactions were performed in a Roche LightCycler 480 (Stratagene, La Jolla, CA, USA), using this program: 2 min at 95°C, 40 cycles of 20 s at 95°C, 20 s at 58°C, and 20 s at 72°C; finally, a melting curve analysis (58°C to 95°C) was conducted to evaluate the specificity of obtained PCR products. Ct values were calculated in Roche qPCR software (v1.5.1) by applying the second derivative method. Each PCR reaction was done in triplicate. Data are presented as the log_2_ (fold-change) in expression and were compared with RNA-Seq results.

## Results

### Overview of Sequencing and ncRNA Predictions

**FIGURE 1:**
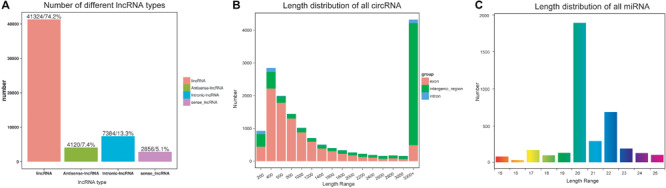
Characteristics of non-coding RNAs in *Dendrolimus punctatus.* **(A)** Classification of the predicted novel lncRNAs; **(B)** length distribution of the circRNAs; **(C)** length distribution of the miRNAs.

In all, 209.24 Gb of clean data with a Q30 > 92.99% were obtained (Supplementary Table S2), and this data mapped onto the *D. punctatus* reference genome assembly, by using HISAT2 (Daehwan et al., 2015; Supplementary Table S3). A total of 55,684 lncRNAs were predicted, most of them being lincRNAs (41,324; 74.2%), followed by intronic-lncRNAs (7,384; 13.3%), antisense-lncRNAs (4,120; 7.4%), and sense lncRNAs (2,856; 5.1%) (Figure 1A). For the lengths of lncRNA and mRNA, they had similar distribution patterns (Supplementary Figures S1A,B) but more of the mRNAs contained four or more exons (Supplementary Figures S1C,D). Most of the ORFs were shorter than 100 bp from the lncRNA, yet longer than 200 bp from the mRNA (Supplementary Figures S1E,F). Additionally, the mRNAs were characterized by more isoforms than were lncRNAs (Supplementary Figure S1G), indicating the former are more complex than the latter. Finally, the expression levels of lncRNAs and mRNAs were generally similar (Supplementary Figure S1H).

Overall, 15,698 circRNAs were predicted in *D. punctatus* according to CIRI, with most of these derived from exons < 3,000 bp whereas most of those derived from intergenic regions were longer than 3,000 bp (Figure 1B). Compared with mRNAs, the expression levels of circRNAs were higher (Supplementary Figure S2).

For the miRNAs, 193.67 Mb of clean reads were acquired, with at least 14.30 Mb per sample, and their Q30 was > 99.43%. From them, any reads less than 15 nt and longer than 35 nt were first removed, the remaining reads were mapped onto the *D. punctatus* reference data (Supplementary Table S4). This yielded 3,738 miRNAs, of which most were between 20 nt and 22 nt (Figure 1C); however, the length distributions of known and novel miRNAs differed (Supplementary Figures S3A,B). The first nucleotide bias analysis indicated that as the total length of a miRNA increased, so did the bias for U serving as the first nucleotide (Supplementary Figure S3C). Additionally, there was evidence of miRNA nucleotide bias at the last position for U and C (Supplementary Figure S3D).

### Expression Differences and Functional Analyses of Coding RNAs Between Low and High Density *D. punctatus*

To analyze the molecular responses of *D. punctatus* during different stages of outbreaks, we compared and selected the DEGs using DESeq (Anders and Huber, 2012). The DEG numbers indicated that the differences between sexes in *D. punctatus* (occurring at both low and high densities) were much greater than those found between densities (both male and female) (Table 1).

**TABLE 1:** Numbers of differentially expressed coding and non-coding RNAs between sexes and population densities of *Dendrolimus punctatus*.

	mRNA			lncRNA			miRNA			circRNA		
DEG Set	all	up	down	all	up	down	all	up	down	all	up	down
Low-♀: Low-♂	6470	3388	3082	473	122	351	581	152	429	77	62	15
High-♀: High-♂	8309	4144	4165	954	417	537	288	40	248	80	55	25
Low-♀: High-♀	561	333	228	138	59	79	347	161	186	39	28	11
Low-♂: High-♂	1371	895	476	169	89	80	239	107	132	81	36	45

*Low, samples from low density; High, samples from high density; all, the number of total DEGs; up, the number of up-regulated DEGs; Down, the number of donw-regulated DEGs.*

**FIGURE 2:**
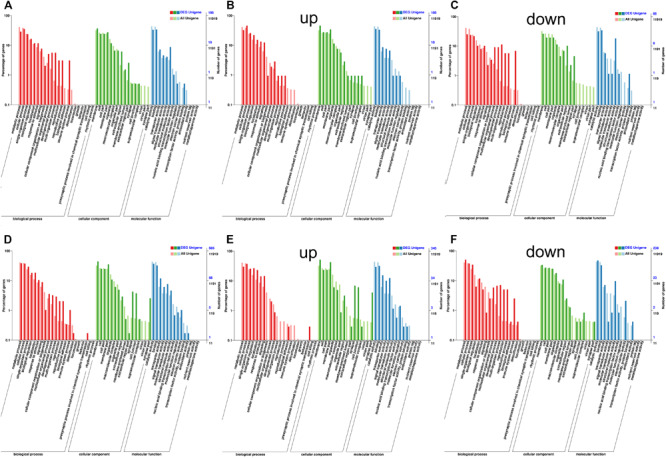
GO annotation of the DEGs (differentially expressed genes) identified between low- vs. high-density *Dendrolimus punctatus*. **(A–C)** For females: total DEGs **(A)**, up-regulated genes **(B)**, and down-regulated genes **(C)**. **(D–F)** For males: total DEGs **(D)**, up-regulated genes **(E)**, and down-regulated genes **(F)**.

Then we focused on analyzing gene expression differences in *D. punctatus* from different outbreak stages. An MA plot of the differences between low- and high-density insects showed the more DEGs in males than females (Supplementary Figures S4A,B). The GO annotation of DEGs in females at low- versus high-density mainly focused on multicellular organism process, developmental process, multi-organism process, reproduction, and immune system process (Figure 2A). The top-10 enriched GO terms for DEGs between low- and high-density female insects revealed that, apart from basic metabolic processes, also included were olfactory-related sensory perception of smell, regulation of postsynaptic membrane potential, chromatin silencing at rDNA (Table 2). These differences indicated that *D. punctatus* females from high-density and low-density populations mainly differ on their development, reproduction, immunity, and olfaction. To detect, in greater detail, further possible differences between low- and high-density insects, we analyzed the up- and down-regulated genes separately. According to their GO enrichment results, this activity was very different between low- and high-density females of *D. punctatus*: the latter’s up-regulated genes were involved in biological regulation, localization, response to stimulus, signaling, developmental process, multi-organism process, and reproduction (Figure 2B); however, the down-regulated genes of these high-density females were involved in multicellular organism process, development process, multi-organism process, reproduction, reproduction process, and immune system process (Figure 2C). Thus, the low-density *D. punctatus* females have better developmental, reproductive, and immune capabilities.

**TABLE 2:** Enriched GO term of DEGs (differentially expressed genes) between low- and high-density female *Dendrolimus punctatus*.

GO.ID	Term	Annotated	Significant	Expected	KS
GO:0006278	RNA-dependent DNA biosynthetic process	331	7	4.9	<1e-30
GO:0090305	nucleic acid phosphodiester bond hydrolysis	342	7	5.06	<1e-30
GO:0015074	DNA integration	90	1	1.33	<1e-30
GO:0090304	nucleic acid metabolic process	1933	25	28.62	4.0e-28
GO:0043551	regulation of phosphatidylinositol 3-kinase activity	27	1	0.4	5.5e-13
GO:0006334	nucleosome assembly	47	1	0.7	1.7e-08
GO:0006259	DNA metabolic process	709	10	10.5	4.8e-05
GO:0050911	detection of chemical stimulus involved in sensory perception of smell	96	0	1.42	0.00098
GO:0060078	regulation of postsynaptic membrane potential	11	3	0.16	0.00106
GO:0000183	chromatin silencing at rDNA	14	1	0.21	0.00312

*Annotated, Annotated gene numbers of the term; Significant, Annotated DEGs numbers of the term; Expected, expected numbers of annotated DEGs numbers of the term; KS, significant statistics of the term.*

For males of *D. punctatus*, a GO term analysis of DEGs between those at low- and high-density revealed they differed significantly in developmental process, multi-organism process, reproduction, and immune system process, as well as the presynaptic process involved in chemical synaptic transmission (Figure 2D). Up- and down-regulated DEGs between low- and high-density males of *D. punctatus* showed different characteristics from the females. The up-regulated genes were involved in biological regulation, localization, response to stimulus, and signaling (much like females), but no developmental and reproductive processes were evidently enriched. Additionally, the presynaptic process involved in chemical synaptic transmission was represented to a higher degree in up-regulated DEGs than among the total genes (Figure 2E). The down-regulated genes were similar to those of females, in that they were mainly involved in multicellular organismal process, developmental process, multi-organism process, reproduction, and immune system process (Figure 2F). These results indicated the low-density males of *D. punctatus* put more energy into development, production, and immune functions than high-density conspecific individuals, while the males at high-density allocate more energy into basic metabolism and olfactory functions.

### Functions of Non-coding RNAs in the Differential Characteristics of Low- and High-Density *D. punctatus*

To analyze the functions of ncRNAs (lncRNA, miRNA, and circRNA) in regulating the outbreak of *D. punctatus*, the differentially expressed ncRNAs were first identified. Using a cutoff of fold change ≥ 2 coupled to an FDR < 0.05, we distinguished 473 and 954 differentially expressed lncRNAs between sexes of *D. punctatus* from low- and high-density populations, respectively; and likewise, 138 and 169 differentially expressed lncRNAs between low- vs. high-density *D. punctatus* in females and males, respectively (Table 1). According to a Volcano plot of DEGs targeted by mRNAs and lncRNAs, the ratio values of differently expressed lncRNAs to mRNAs between low- and high-density areas (Supplementary Figures S5A,B) was higher than those between females and males (Supplementary Figures S5C,D); this indicated a stronger regulatory role of lncRNAs in generating differences found between low- and high-density insects than those between sexes. Applying the same cutoff criteria as above, 77 and 80 differentially expressed circRNAs between sexes of *D. punctatus* from low- and high-density areas, and 39 and 81 differentially expressed circRNAs between low- vs. high-density *D. punctatus* in females and males were identified, respectively (Table 1). Likewise, for differentially expressed miRNAs, 581 and 288 of them were found between female and male *D. punctatus* from low- and high-density populations, respectively, with 347 and 239 identified between low- vs. high-density *D. punctatus* in the females and males, respectively (Table 1).

**FIGURE 3:**
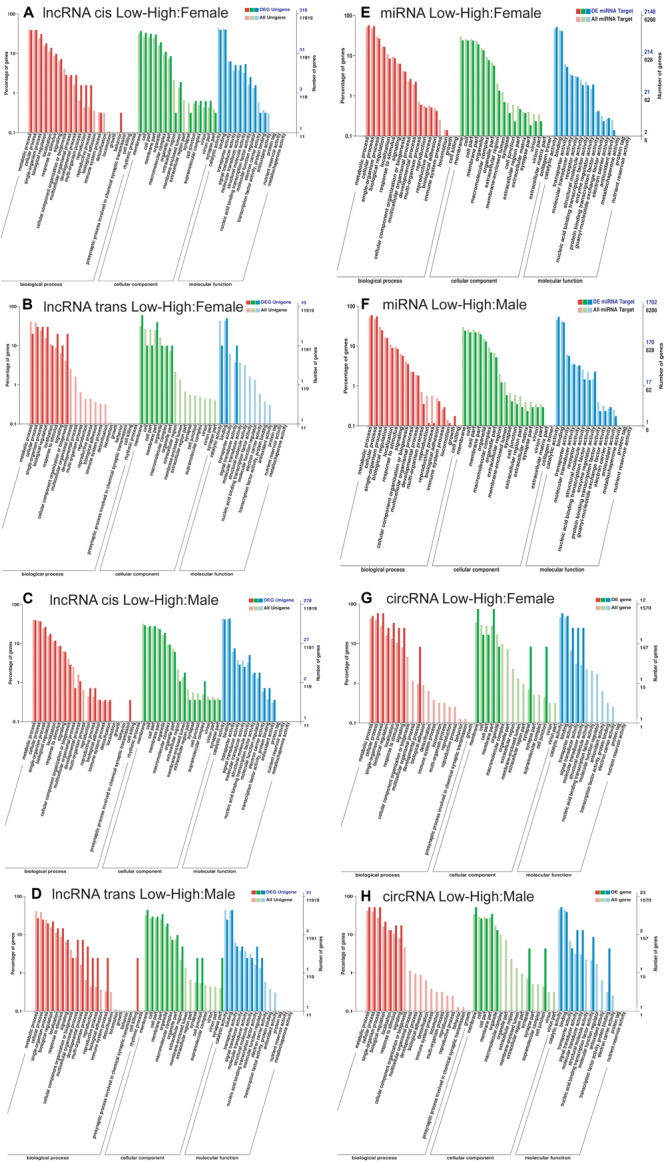
GO annotation of the targets of differently expressed non-coding RNAs identified between low- vs. high-density *Dendrolimus punctatus*. **(A–D)** lncRNAs: cis **(A)** and trans **(B)** GO annotations of differentially expressed lncRNAs between *D. punctatus* females from the low- and high-density populations. Likewise, cis **(C)** and trans **(D)** GO annotations of the differentially expressed lncRNAs between *D. punctatus* males from the low- and high-density populations. **(E,F)** miRNAs: GO annotation of the targets of differentially expressed miRNAs between low- vs. high-densities in females **(E)** and males **(F)**. **(G,H)** circRNAs: GO annotation of genes targeted by the differentially expressed circRNAs between low- vs. high-densities in females **(G)** and males **(H)**.

Next, the targets of differently expressed ncRNAs between low- and high-density *D. punctatus* insects were further analyzed to explore the regulating roles of ncRNAs during this insect’s outbreaks. The lncRNAs may regulate the associated mRNA genes of neighboring (cis target) and overlapping (trans targets) (Li et al., 2017a). Hence, both the cis and trans targets of lncRNAs were predicted and analyzed. For females, the cis targets of differentially expressed lncRNA between low- and high-density insects were mainly involved in developmental and multi-organism processes, reproduction, and behavior, while the trans targets were involved in biological regulation, response to stimulus, signaling, and cellular component organization or biogenesis (Figures 3A,B). The differently expressed mRNAs (Figure 2A) and the targets of differently expressed lncRNAs between low- and high-density females of *D. punctatus* were both involved in metabolism, development, and reproduction; however, their enriched GO terms revealed some divergence, in that mRNAs were more specific to the immunity term, whereas lncRNAs’ targets were more specific to the behavior term. For males, the cis targets of differentially expressed lncRNAs between low- and high-density insects were mainly involved in reproduction and the presynaptic process involved in chemical synaptic transmission, while the trans targets participated in multicellular organismal, developmental, and immune system processes, as well as reproduction and cell killing (Figures 3C,D). In sum, the GO terms of differently expressed mRNAs and targets of differently expressed lncRNAs from male insects at low- and high-density were coincident, indicating a primary regulator role for lncRNA in *D. punctatus* outbreaks.

The respective functions of miRNAs related the differing characteristics of low- and high-density populations of *D. punctatus* were then analyzed. In females, there were fewer target genes of differentially expressed miRNAs between low- and high-density *D. punctatus* than targets of all the miRNAs in the immune system process (Figure 3E). Because miRNAs play negative regulatory roles on their target genes (Ghildiyal and Zamore, 2009), these results indicated that miRNA play more regulatory roles in the low-density insect. In males, fewer target genes of differentially expressed miRNAs between low- and high- density *D. punctatus* populations were found than targets of all the miRNAs in both the multi-organism process and reproduction terms (Figure 3F). This indicated that these miRNAs function in metabolic and reproductive adjustments that occur between low- and high-density males of *D. punctatus*.

The targeted genes of differentially expressed circRNAs between low- and high-density populations of *D. punctatus* were also analyzed. In females, these mainly participated in the biological process category of development, the cellular component category of synapse and cell junction, and the molecular function categories of transporter, signal transducer, and molecular transducer activities (Figure 3G). In males, they were primarily involved in the cellular component category of synapse and cell junction, as well as molecular function categories of protein binding and signal transducer, molecular transducer, nucleic acid-binding transcription factor, and transcription factor activities (Figure 3H). These results revealed the different targeted genes of the differentially expressed circRNAs; interestingly, the category synapse and cell junction was enriched in both comparisons.

To verify the RNA-Seq data, we selected thirteen mRNA, three lncRNA, three miRNA, and three circRNA randomly. Their respective fold-change values in expression between low-and high-density *D. punctatus* were tested by qRT-PCR. These results verified the reliability of the initial set of RNA-Seq results (Supplementary Figure S6).

### Construction of ceRNA Networks in *D. punctatus*

**FIGURE 4:**
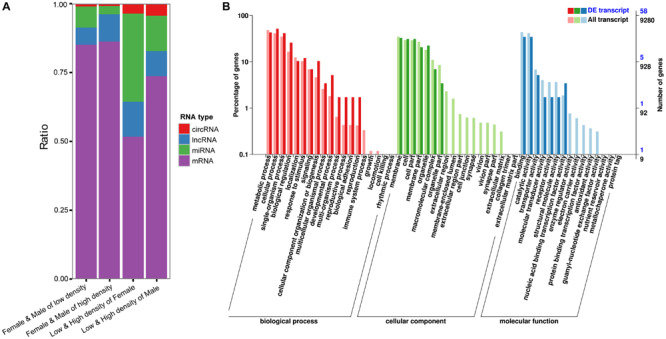
**(A)** Total numbers of differentially expressed coding and ncRNAs in four situations tested; **(B)**. GO annotation of critical genes in the ceRNA network.

The circRNAs, lncRNAs, and mRNAs may bind to the same miRNA response elements competitively in a regulatory network, which is referred to as a ceRNA network (Salmena et al., 2011). Figure 4A shows the total numbers of differentially expressed coding and ncRNAs in different situations. The ratios of ncRNAs targeting DEGs between low- and high-density populations are higher than those between males and females, indicating the regulatory functions of ncRNAs were stronger in the outbreak process than between sexes. Then, to derive a ceRNA network, we screened the relationships between miRNA vs. mRNA, miRNAs vs. circRNA and miRNAs vs. lncRNA (Supplementary Table S5), using the following parameters: number of miRNAs interacting with the candidate ceRNAs > 5 and P < 0.05 (Li et al., 2014). From this, a total of 55,707,481 ceRNA relationships with 41,508 nodes were obtained. With such a complex network, it was necessary to extract the critical RNAs and perform a follow-up analysis: score for all the nodes in the network were obtained and the top 10% of nodes were kept for further examination. A GO annotation of these critical RNAs indicated their involvement in developmental, multi-organism, and reproduction processes, as well as biological adhesion (Figure 4B), and these GO terms also figured prominently among the DEGs distinguished between low- and high-density *D. punctatus* populations. Taken together, these results indicated that an operating ceRNA regulatory network is crucial during insect outbreaks.

### Differently Expressed Chemosensory and Immune Genes Between Low- and High-Density *D. punctatus* and Their ncRNA Regulators

**FIGURE 5:**
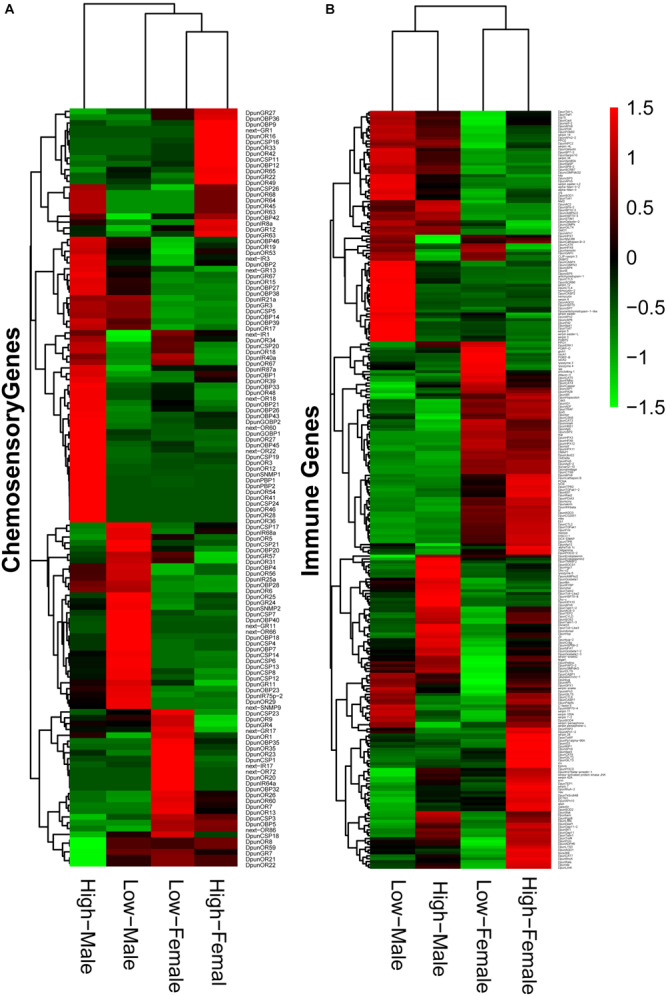
Expression of chemosensory genes **(A)** and immune genes **(B)** in male and female *Dendrolimus punctatus* from low- and high-density populations.

The chemosensory and immune system closely related to the physiological characteristics during different outbreak stages of *D. punctatus*, as indicated in the previous research (Billings, 1991; Zhang et al., 2002). Our transcriptomic results also illustrated their critical roles in the outbreak process of this defoliating insect. Accordingly, we further analyzed these two gene families in low- and high-density *D. punctatus*. Heatmaps (Figure 5) showed that relative expression levels of chemosensory and immune genes not only differed greatly between sexes, but also between individuals at low-and high-density, especially in male insects.

**FIGURE 6:**
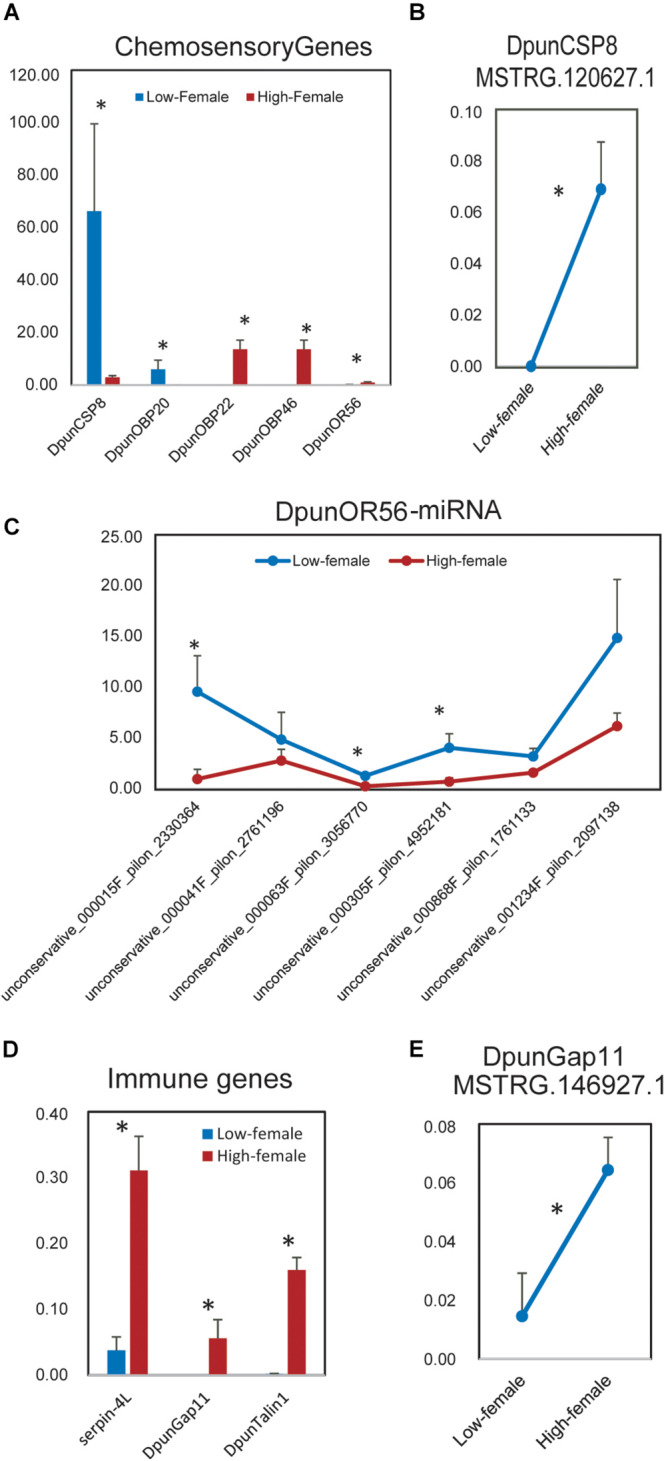
Differently expressed chemosensory and immune genes and their significant differently expressed non-coding RNA regulators between low- vs. high-density females of *Dendrolimus punctatus*. **(A)** Differently expressed chemosensory genes; **(B)** significant differently expressed lncRNA regulator of *DpunCSP8*; **(C)** miRNA regulators of DpunOR56; **(D)** differently expressed immune genes; **(E)** significant differently expressed lncRNA regulator of DpunGap11.

Then, we investigated in depth the differently expressed chemosensory and immune genes between low- and high-density *D. punctatus* and their ncRNA regulators. In females, the expression of five chemosensory genes was statistically different between low- and high-density *D. punctatus* (Figure 6A): it was greater for *DpunCSP8* and *DpunOBP20* in low-density females, but greater for *DpunOBP22*, *DpunOBP46*, and *DpunOR56* in high-density females. 14 lncRNAs contained *DpuncCSP8* as their target (Supplementary Figure S7A), yet only MSTRG.120627.1 was expressed significantly higher in high-density females (Figure 6B). Eight, six, and six lncRNAs respectively contained *DpuncOBP20*, *DpuncOBP22*, and *DpuncOBP46* as targets (Supplementary Figures S7B–D), but none of these lncRNAs were expressed in a significantly different way between low- and high-density females. Six miRNAs contained *DpuncOR56* as their target, of which three were significantly down-regulated in females at high-density (Figure 6C, Supplementary Figure S7E), the reverse of *DpunOR56*’s expression. Three immune genes, *Serpin-4L*, *DpunGap11*, and *DpunTalin1*, were found expressed significantly higher in high-density than in low-density females of *D. punctatus* (Figure 6D). No ncRNA regulator could be found for Serpin-4L. Eight lncRNAs contained *DpunGap11* as a target (Supplementary Figure S7F), with only MSTRG.146927.1 expressed at a significantly higher level in the high-density than low-density females (Figure 6E). *DpunTalin1* was target of one lncRNA (Supplementary Figure S7G), one miRNA (Supplementary Figure S7H), and seven circRNAs (Supplementary Figure S7I); however, none of these ncRNAs were expressed in a significantly different way between low- and high-density females.

**FIGURE 7:**
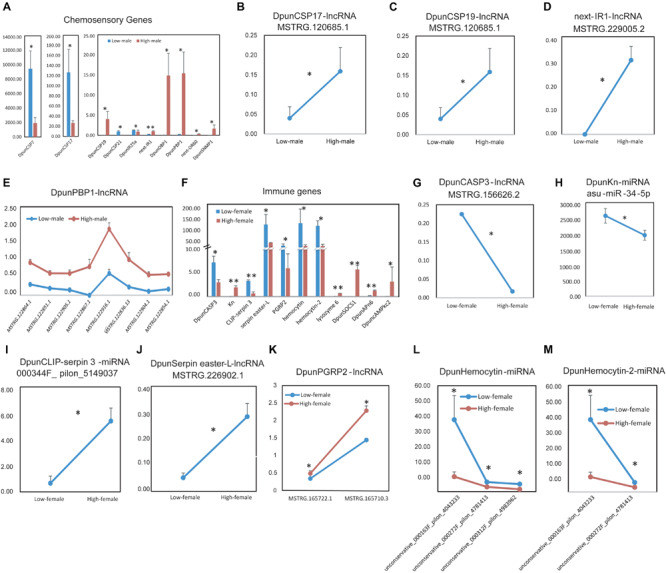
Differently expressed chemosensory and immune genes and their significant differently expressed non-coding RNA regulators between low- vs. high-density males of *Dendrolimus punctatus*. **(A)** Differently expressed chemosensory genes; **(B–E)** significant differently expressed non-coding RNA regulators of chemosensory genes in **(A)**. **(F)** Differently expressed immune genes; **(G–M)** significant differently expressed non-coding RNA regulators of immune genes in **(F)**.

Next, we considered the situations of low- vs. high-density males of *D. punctatus*, finding more differently expressed chemosensory or immune genes in males than females between low- and high-density populations of *D. punctatus* (Figure 7). Ten chemosensory genes were expressed at significantly differently levels between low- and high-density males of *D. punctatus* (Figure 7A): *DpunCSP7*, *Dpund17*, *DpunIR21*, and *DpunIR25a* were expressed more in low-density males, whereas *DpunCSP19*, *DpunOBP1*, *DpunPBP1*, *next-OR60*, and *DpunSNMP1* were all expressed more in high-density males. Among these chemosensory genes, an lncRNA regulator, MSTRG.120685.1, of both *DpunCSP17* (Figure 7B) and *DpunCSP19* (Figure 7C), had significantly more expression in high-density males, as did another lncRNA regulator, MSTRG.120685.1, of *next-IR1* (Figure 7D); *DpunPBP1* was regulated by 30 lncRNAs (Figure S8E), of which eight were expressed significantly more in high-density males (Figure 7E). The ncRNA regulators of other chemosensory genes did not undergo a significantly different level of expression between low- and high-density males of *D. punctatus* (Supplementary Figure S8). Eleven immune genes were expressed significantly and differently between low- and high-density male *D. punctatus* (Figure 7F), of which six were down-regulated and five were up-regulated in high-density males. Among these immune genes, the lncRNA regulator, MSTRG.156626.2, of *DpunCASP3* (Figure 7G), was expressed significantly more in low-density males, as was the miRNA regulator, asu-miR-34-5p, of *DpunKn* (Figure 7H). Similarly, another miRNA regulator, unconservative_000344F_pilon_5149037, of *DpunCLIP-serpin 3* (Figure 7I), had significantly greater expression in high-density males, as did the lncRNA regulator MSTRG.226902.1 of *DpunSerpin easter-L* (Figure 7J) and both lncRNA regulators, MSTRG.165722.1 and MSTRG.165710.3, of *DpunPGRP2* (Figure 7K). Finally, *DpunHemocytin* and *DpunHemocytin-2* were both regulated by a miRNA that was expressed significantly higher in low-density males (Figures 7L,M). The ncRNA regulators of other immune genes did show any significantly expressed differences between low- and high-density males of *D. punctatus* (Supplementary Figure S9).

## Discussion

Insect species that experience outbreaks undergo extreme fluctuations in population density and consequently are capable of exerting substantial ecological and economic effects over large areas (Baltensweiler and Fischlin, 1988; Pener and Simpson, 2009). Understanding the large-scale and long-term dynamic mechanisms of outbreaking insects is therefore necessary to develop effective management strategies to mitigate these occurrences. Hence, in this study we employed whole-transcriptome sequencing and bioinformatics technology to comprehensively analyze the outbreak-related functions of coding and ncRNAs profiles and ceRNA networks in a typical forest insect pest, the defoliating moth *D. punctatus*.

Currently, molecular mechanism-related studies of *D. punctatus* outbreak characteristics remain limited to mRNAs (Yang et al., 2016; Yang C.H. et al., 2017; Zhang et al., 2016, 2018), while the study of ncRNAs in forest insects is generally limited. Our study is the first to identify and quantify expression levels of lncRNA, circRNA, and miRNA in *D. punctatus*. We identified 55,684 novel lncRNAs, in addition to 5,698 novel circRNAs and 3,521 novel miRNAs. Furthermore, differentially expressed coding and non-coding RNAs were identified between the low- and high-density *D. punctatus* populations, for which functional annotations revealed their specific regulatory roles in this pest’s outbreaks.

Firstly, the differentially expressed mRNAs related to *D. punctatus* population density were investigated. The differences arising between low- and high-population densities in males and females were various. In females, the DEGs were mainly involved in metabolism, development, reproduction and immunity, while in males, in addition to metabolism, development, reproduction, and immunity, the DEGs contributed to synapse junctions. Reproduction and immunity differences between low and high density of both sexes are consistent with other research in which fertility and parasitism rates differed during the non-outbreak and outbreak stages of *D. punctatus* (Zhang et al., 2002). The synapse is involved in neuron-related activities, such as learning and memory (Priyamvada et al., 2009; Fiumara et al., 2015). We posit that the differences in synaptic capabilities found between low- and high-density male insects reflect their varied neural activities. In this way, the molecular basis of differences in physiological traits of low- and high-density *D. punctatus* could be outlined.

The ncRNAs appear to play important roles in cellular functions, particularly in organismal responses to biotic and abiotic stresses (Gomes et al., 2013), and our results suggest the regulatory functions of ncRNAs in insect outbreaks are also significant. The lncRNAs participate in the regulation of different biological processes, albeit in disparate ways (Long et al., 2017; Marchese et al., 2017). Our results indicate that lncRNAs have regulatory functions during the outbreaking process of *D. punctatus*. In females of *D. punctatus*, the targets of differentially expressed lncRNAs between low- and high-density populations were mostly involved in metabolism, development, reproduction, and behavior. These functions are similar to those of their differently expressed mRNAs. Additionally, in males, the functions of the targets of differentially expressed lncRNAs were consistent with those of differently expressed mRNAs. Consequently, we conclude that lncRNAs are the primary ncRNA regulators of molecular differences that arise in this insect when its population is at low versus high density. The regulatory functions of lncRNAs during insect reproduction have been demonstrated (Wen et al., 2016; Maeda et al., 2018), and our work here indicates that reproductive regulation of lncRNAs can also affect different stages of insect outbreaks. Immune-related regulatory functions of lncRNAs are extensively researched in animals and humans (Atianand et al., 2017; Chen et al., 2017; Mowel et al., 2018), but a focus on insects has been limited. Our results demonstrated that immune-related regulatory functions of lncRNAs also operate in insects, and this may, in part, explain the resulting different immune capabilities of low- and high-density individuals of *D. punctatus*.

The circRNA revealed the existence of different regulatory functions between sexes in low- and high-density *D. punctatus* populations, but they both participated in synapse and cell junction. This result is consistent with previous findings in which synapse regulation was determined to be a vital function of circRNAs (Westholm et al., 2014; You et al., 2015). As newly found ncRNAs, research on the functioning of circRNAs remains limited to humans and a few vertebrate species (Li et al., 2017b; Xiu et al., 2019; Zhang et al., 2019). The regulatory functions of circRNAs in insects during changes in their population density deserve further research.

Being important epigenetic regulators of mRNA translation, miRNAs’ involvement is implicated in many critical biological processes, such as learning, reproduction, development, metabolism, immunity, and aging (Priyamvada et al., 2009; Nan et al., 2012; Fiumara et al., 2015; Mehta and Baltimore, 2016; Ling et al., 2017; Meuti et al., 2018; Roy et al., 2018). However, miRNAs often operate in networks (Guo et al., 2016), and genome-wide analyses of miRNAs can help to reveal the complexity inherent to these networks (Li Y. et al., 2017). Our whole-transcriptome sequencing of both coding and non-coding RNAs could facilitate the identification of miRNA and miRNA–mRNA interacting pairs in *D. punctatus*. Functional annotations of the target genes of differentially expressed miRNAs indicated they are chiefly involved in regulating the immune and reproductive pathways during outbreaks of *D. punctatus*. Further functional analyses are essential to confirm the roles of miRNAs in insect outbreaks.

The “ceRNA hypothesis,” proposed by Salmena et al. (2011), describes how lncRNAs or circRNAs could inhibit miRNAs to positively regulate targeted mRNAs, by unifying the functions of several coding and ncRNAs. No ceRNAs were previously reported in *D. punctatus*, and so here we analyzed the ceRNA network related to *D. punctatus* outbreaks for the first time. Key genes analysis of the *D. punctatus* population density-related ceRNA network indicated that the developmental process, multi-organism process, reproduction process, and biological adhesion were components of its ceRNA network. These findings provide a theoretical basis for further investigations of the outbreak-related mechanisms of *D. punctatus* and other similar insects.

Besides the basic metabolic processes, our results indicated that chemosensory and immune genes play important roles in outbreak of *D. punctatus*. We found that chemosensory and immune genes that were differently expressed between low- and high-density *D. punctatus* characterized the males more than females. The lncRNAs were the main regulators for these DEGs, yet miRNA regulators were also found in several of these genes. Most of these lncRNA regulators had the same expression pattern as their mRNA targets, but some of them also showed reverse expression patterns vis-à-vis their mRNA targets, such as lncRNA MSTRG.120685.1 and *DpunCSP17*. These results suggest a complex regulation effect imparted by lncRNAs (Rinn and Chang, 2012). miRNA can regulate the olfactory activities of insects (Guo et al., 2018), and we also found that olfactory receptor was regulated by miRNAs in *D. punctatus*; but the regulatory roles of miRNA for immune gene were more comment in *D. punctatus*. Most of these miRNAs’ expression patterns were the opposite of their mRNA targets, a finding consistent with other research (Ghildiyal and Zamore, 2009). Nevertheless, some unexpected cases were also found in our study. For example, the expression levels of miRNA regulators of *DpunHemocytin* and *DpunHemocytin-2* as well as those these two genes were all down-regulated in the high-density population of *D. punctatus* insects. The reasons for these unexpected results may be inaccurate target predictions (Riffo-Campos et al., 2016) or that the miRNA acts as a subsidiary regulators of these genes, highlighting the need for further mechanistic research. Our work indicates that ncRNAs are crucial regulators during outbreaks of *D. punctatus*, but the mechanisms underpinning interactions between differently expressed genes and their ncRNA regulators merits further investigation.

## Conclusion

In this study, we elucidated the coding and non-coding profiles of control and outbreak insects from a whole-transcriptome analysis. The results indicated that the physiological differences between low- and high-density *D. punctatus* matched well to the functions of identified DEGs. Accordingly, we may deduce that reproduction, immunity, and multi-organism processes, and the chemosensory system, are the key signal pathways involved in this insect’s outbreaks. Analysis of three kinds of ncRNAs suggested that lncRNAs are the primary regulators, while miRNAs function mainly in the immune and reproduction adjustments made at different outbreak stages in *D. punctatus*. Further analysis of those chemosensory and immune genes closely related to outbreaking *D. punctatus* showed that its males contain more DEGs than do females between low- and high-density insects, and by analyzing their non-coding RNA regulators we deduced that lncRNA and miRNA play important roles during the outbreak process. To the best of our knowledge, this is the first report to have examined lncRNAs, circRNAs, miRNAs, and mRNAs expression levels and their functions in an outbreaking insect species. These findings increase our understanding of ceRNA networks and can generally aid and guide further exploration of their regulatory functions in key physiological processes required for insect outbreaks to emerge. However, the outbreak of an insect population is a complicated process: the regulatory relationships among the factors we identified above, along with initiating factors of this process, remain unclear, and so further functional-related research on these key DEGs and their non-coding regulators is necessary. Our work provides new and timely insights into the mechanisms underlying insect outbreaks that could inform control strategies to mitigate them.

## Data Availability Statement

Raw reads from sequencing are deposited in the Sequence Read Archive (SRA) database with NCBI accessiona SRR10481893–SRR10481916.

## Author Contributions

ZZ and SZ designed the research. SZ, SS, ZY, XK, and FL, collated the sample, performed the research and/or analyzed the data. SZ wrote the manuscript. ZZ revised the manuscript. All authors reviewed the manuscript.

## Conflict of Interest

The authors declare that the research was conducted in the absence of any commercial or financial relationships that could be construed as a potential conflict of interest.

## Footnotes

1 https://ccb.jhu.edu/software/stringtie/index.shtml

2 http://ccb.jhu.edu/software/stringtie/gffcompare.shtml

3 http://www.mirbase.org/

## Supplementary Material

FIGURE S1 Structural features and expression comparisons of lncRNAs and mRNAs in *Dendrolimus punctatus*. **(A)** Lengths of lncRNAs; **(B)** lengths of mRNAs; **(C)** exon numbers of lncRNAs; **(D)** exon numbers of mRNAs; **(E)** ORF lengths of lncRNAs; **(F)** ORF lengths of mRNAs; **(G)** the alternatively spliced isoforms of lncRNAs and mRNAs; **(H)** the expression levels of lncRNAs and mRNAs.

FIGURE S2 FPKM comparisons for the mRNAs and circRNAs.

FIGURE S3 Characterization of miRNAs in *Dendrolimus punctatus*. **(A,B)** Length distributions of known **(A)** and novel **(B)** miRNAs identified in this study; **(C)** first nucleotide bias of miRNAs; **(D)** nucleotide bias analysis at each miRNA position.

FIGURE S4 MA plot of the differences between low- and high-density *Dendrolimus punctatus* in females **(A)** and males **(B)**.

FIGURE S5 Volcano plot of mRNA and lncRNA-targeted DEGs in *Dendrolimus punctatus* between low- vs. high-density population in females **(A)**, males **(B)**, and between sexes in low- **(C)** and high- **(D)** density populations.

FIGURE S6 Real-time PCR validation of the RNA-Seq data. The *x*-axis shows the RNA names, and the *y*-axis is their log_2_ (fold change) based on the ratio of high-density and low-density insects.

FIGURE S7 All non-coding RNA regulators of differently expressed chemosensory and immune genes between low- vs. high-density females of *Dendrolimus punctatus*. **(A–D)** lncRNA regulators of *DpunCSP8*, *DpunOBP20*, *DpunOBP22*, *DpunOBP46*; **(E)** miRNA regulators of *DpunOR56*; **(F)** lncRNA regulators of *DpunGap11*; **(G–I)** lncRNA, miRNA, and circRNA regulators of *DpunTalin1*.

FIGURE S8 All non-coding RNA regulators of differently expressed chemosensory genes between low- vs. high-density males of *Dendrolimus punctatus*. **(A,B)** lncRNA and circRNA regulators of *DpunCSP7*; **(C–F)** lncRNA regulators of *DpunCSP17*, *DpunCSP19*, *DpunCSP21*, and *DpunIR25a*; **(G,H)** lncRNA and miRNA regulators of *DpunNext-IR1*; **(I–K)** lncRNA regulators of *DpunPBP1*, *DpunNext-OR60*, and *DpunSNPM1*.

FIGURE S9 All non-coding RNA regulators of differently expressed immune genes between low- vs. high-density males of *Dendrolimus punctatus*. **(A,B)** lncRNA and circRNA regulators of *DpunCASP3*; **(C,D)** lncRNA and miRNA regulators of *DpunKn*; **(E,F)** lncRNA and miRNA regulators of *DpunCLIP-serpin* 3; **(G,H)** lncRNA regulators of *DpunSerpin easter-L* and *DpunPGRP2*; **(I)** miRNA regulators of *DpunHemocytin*; **(J,K)** lncRNA and miRNA regulators of *DpunHemocytin-2*; **(L,M)** lncRNA and circRNA regulators of *DpunAPn6*; **(N,O)** lncRNA and miRNA regulators of *DpuncAMPkc2*.

TABLE S1 Primers used for the gene expression validation with Real-time PCR.

TABLE S2 Summary of the sequenced data for *D. punctatus*.

TABLE S3 Sequence alignment results for the comparison of sequencing data to selected reference genomes.

TABLE S4 Sequence alignment results for the comparison of sequenced miRNAs to selected reference genomes.

TABLE S5 circRNA, lncRNA, and mRNA gene numbers that correlated to miRNA.

## References

[B1] Alexa A. Rahnenfuhrer J. (2010). *topGO: Enrichment Analysis for Gene Ontology. R Package (Version 2.18 ed.).*

[B2] Alison F. H. (1991). Traits that distinguish outbreaking and nonoutbreaking macrolepidoptera feeding on northern hardwood trees. *Oikos* 60 275–282. 10.2307/3545068

[B3] Amaral P. P. Dinger M. E. Mercer T. R. Mattick J. S. (2008). The eukaryotic genome as an RNA machine. *Science* 319:1787. 10.1126/science.1155472 18369136

[B4] Anders S. Huber W. (2012). *Differential Expression of RNA-Seq Data At The Gene Level - the DESeq Package”. R Package.*

[B5] Atianand M. K. Caffrey D. R. Fitzgerald K. A. (2017). Immunobiology of long noncoding RNAs. *Ann. Rev. Immunol.* 35 177–198. 10.1146/annurev-immunol-041015-055459 28125358 PMC6449690

[B6] Baltensweiler W. Fischlin A. (1988). “The larch budmoth in the alps,” in *Dynamics of Forest Insect Populations. Population Ecology (Theory and Application)*, ed. Berryman A. A. (Boston, MA: Springer).

[B7] Beermann J. Piccoli M.-T. Viereck J. Thum T. (2016). Non-coding RNAs in development and disease: background, mechanisms, and therapeutic approaches. *Physiol. Rev.* 96 1297–1325. 10.1152/physrev.00041.2015 27535639

[B8] Belles X. (2017). MicroRNAs and the evolution of insect metamorphosis. *Annu. Rev. Entomol.* 62 111–125. 10.1146/annurev-ento-031616-034925 27813669

[B9] Berryman A. A. (1988). *Dynamics of Forest Insect Populations: Patterns, Causes, Implications.* New York, NY: Plenum Press.

[B10] Berryman A. A. Stenseth N. C. Isaev A. S. (1987). Natural regulation of herbivorous forest insect populations. *Oecologia* 71 174–184. 10.1007/BF00377282 28312243

[B11] Billings R. F. (1991). The pine caterpillar *Dendrolimus punctatus* in viet nam; recommendations for integrated pest management. *Forest Ecol. Manag.* 39 97–106. 10.1016/0378-1127(91)90167-T

[B12] Chen B. Zhang Y. Zhang X. Jia S. Chen S. Kang L. (2016). Genome-wide identification and developmental expression profiling of long noncoding RNAs during *Drosophila metamorphosis*. *Sci. Rep.* 6:23330. 10.1038/srep23330 26996731 PMC4800424

[B13] Chen C. (ed.). (1990). “Species, geographic distributions, and biological characteristics of pine caterpillars in China,” in *Integrated Management of Pine Caterpillars in China*, (Beijing: China Forestry Publishing House), 5–18.

[B14] Chen Y. G. Satpathy A. T. Chang H. Y. (2017). Gene regulation in the immune system by long noncoding RNAs. *Nat. Immunol.* 18 962–972. 10.1038/ni.3771 28829444 PMC9830650

[B15] Costa F. F. (2005). Non-coding RNAs: new players in eukaryotic biology. *Gene* 357 83–94. 10.1016/j.gene.2005.06.019 16111837

[B16] Daehwan K. Ben L. Salzberg S. L. (2015). HISAT: a fast spliced aligner with low memory requirements. *Nat. Methods* 12 357–360. 10.1038/nmeth.3317 25751142 PMC4655817

[B17] Doron B. Manda W. Aaron G. Marks D. S. Chris S. (2008). The microRNA.org resource: targets and expression. *Nucleic Acids Res.* 36 149–153. 10.1093/nar/gkm995 PMC2238905 18158296

[B18] Elderd B. D. Rehill B. J. Haynes K. J. Dwyer G. (2013). Induced plant defenses, host-pathogen interactions, and forest insect outbreaks. *Proc. Natl. Acad. Sci. U.S.A.* 110 14978–14983. 10.1073/pnas.1300759110 23966566 PMC3773759

[B19] Fahlgren N. Howell M. D. Kasschau K. D. Chapman E. J. Sullivan C. M. Cumbie J. S. (2007). High-throughput sequencing of *Arabidopsis* microRNAs: evidence for frequent birth and death of MIRNA genes. *PLoS One* 2:e219. 10.1371/journal.pone.0000219 17299599 PMC1790633

[B20] Finn R. D. Alex B. Jody C. Penelope C. Eberhardt R. Y. Eddy S. R. (2014). Pfam: the protein families database. *Nucleic Acids Res.* 42 222–230. 10.1093/nar/gkt1223 PMC3965110 24288371

[B21] Fiumara F. Rajasethupathy P. Antonov I. Kosmidis S. Sossin W. S. Kandel E. R. (2015). MicroRNA-22 gates long-term heterosynaptic plasticity in *Aplysia* through presynaptic regulation of CPEB and downstream targets. *Cell Rep.* 11 1866–1875. 10.1016/j.celrep.2015.05.034 26095361

[B22] Friedländer M. R. Mackowiak S. D. Li N. Chen W. Rajewsky N. (2012). miRDeep2 accurately identifies known and hundreds of novel microRNA genes in seven animal clades. *Nucleic Acids Res.* 40 37–52. 10.1093/nar/gkr688 21911355 PMC3245920

[B23] Fuentealba A. Pureswaran D. Bauce E. Despland E. (2017). How does synchrony with host plant affect the performance of an outbreaking insect defoliator? *Oecologia* 184 847–857. 10.1007/s00442-017-3914-3914 28756489

[B24] Ghildiyal M. Zamore P. D. (2009). Small silencing RNAs: an expanding universe. *Nat. Rev. Genet.* 10 94–108. 10.1038/nrg2504 19148191 PMC2724769

[B25] Gomes A. Q. Nolasco S. Soares H. (2013). Non-coding RNAs: multi-tasking molecules in the cell. *Intern. J. Mol. Sci.* 14 16010–16039. 10.3390/ijms140816010 23912238 PMC3759897

[B26] Guo W. Wang X. Ma Z. Xue L. Han J. Yu D. (2011). CSP and takeout genes modulate the switch between attraction and repulsion during behavioral phase change in the *Migratory locust*. *PLoS Genet.* 7:e1001291. 10.1371/journal.pgen.1001291 21304893 PMC3033386

[B27] Guo X. Ma Z. Du B. Li T. Li W. Xu L. (2018). Dop1 enhances conspecific olfactory attraction by inhibiting miR-9a maturation in locusts. *Nat. Commun.* 9:1193. 10.1038/s41467-018-03437-z 29567955 PMC5864846

[B28] Guo Y. Alexander K. Clark A. G. Grimson A. Yu H. (2016). Integrated network analysis reveals distinct regulatory roles of transcription factors and microRNAs. *RNA* 22 1663–1672. 10.1261/rna.048025.114 27604961 PMC5066619

[B29] Hansen T. B. Jensen T. I. Clausen B. H. Bramsen J. B. Finsen B. Damgaard C. K. (2013). Natural RNA circles function as efficient microRNA sponges. *Nature* 495 384–388. 10.1038/nature11993 23446346

[B30] Haynes W. (2013). “Benjamini-hochberg method,” in *Encyclopedia of Systems Biology*, eds Dubitzky W. Wolkenhauer O. Cho K.-H. Yokota H. (New York, NY: Springer), 78–78. 10.1007/978-1-4419-9863-7_1215

[B31] He J. Chen Q. Wei Y. Jiang F. Yang M. Hao S. (2016). MicroRNA-276 promotes egg-hatching synchrony by up-regulating *brm* in locusts. *Proc. Natl. Acad. Sci. U.S.A.* 113 584–589. 10.1073/pnas.1521098113 26729868 PMC4725505

[B32] Hombach S. Kretz M. (2016). “Non-coding RNAs: classification, biology and functioning,” in *Non-Coding RNAs in Colorectal Cancer*, eds Slaby O. Calin G. A. (Cham: Springer International Publishing), 3–17. 10.1007/978-3-319-42059-2_1 27573892

[B33] Hunter A. F. (1995). “Chapter 3 - ecology, life history, and phylogeny of outbreak and nonoutbreak species,” in *Population Dynamics*, eds Cappuccino N. Price P. W. (San Diego: Academic Press), 41–64. 10.1016/b978-012159270-7/50004-x

[B34] Jeck W. R. Sharpless N. E. (2014). Detecting and characterizing circular RNAs. *Nat. Biotechnol.* 32 453–461. 10.1038/nbt.2890 24811520 PMC4121655

[B35] Kai C. Lei W. Xu C. Zhang X. Xing L. Zuo H. (2013). Influence on surgical treatment of intertrochanteric fracture with or without preoperative skeletal traction. *Nucleic Acids Res.* 41:e74.

[B36] Kang L. Chen X. Zhou Y. Liu B. Zheng W. Li R. (2004). The analysis of large-scale gene expression correlated to the phase changes of the migratory locust. *Proc. Natl. Acad. Sci. U.S.A.* 101 17611–17615. 10.1073/pnas.0407753101 15591108 PMC535406

[B37] Kelley D. Rinn J. (2012). Transposable elements reveal a stem cell-specific class of long noncoding RNAs. *Genome Biol.* 13:R107. 10.1186/gb-2012-13-11-r107 PMC3580499 23181609

[B38] Kretz M. Webster D. E. Flockhart R. J. Lee C. S. Zehnder A. Lopez-Pajares V. (2012). Suppression of progenitor differentiation requires the long noncoding RNA ANCR. *Genes Dev.* 26:338. 10.1101/gad.182121.111 22302877 PMC3289881

[B39] Langmead B. Salzberg S. L. (2012). Fast gapped-read alignment with Bowtie 2. *Nat. Methods* 9 357–359. 10.1038/nmeth.1923 22388286 PMC3322381

[B40] Lei K. Yong Z. Zhi-Qiang Y. Xiao-Qiao L. Shu-Qi Z. Liping W. (2007). CPC: assess the protein-coding potential of transcripts using sequence features and support vector machine. *Nucleic Acids Res.* 35:W345. 10.1093/nar/gkm391 PMC1933232 17631615

[B41] Li J.-H. Liu S. Zhou H. Qu L.-H. Yang J.-H. (2014). starBase v2.0: decoding miRNA-ceRNA, miRNA-ncRNA and protein-RNA interaction networks from large-scale CLIP-Seq data. *Nucleic Acids Res.* 42 D92–D97. 10.1093/nar/gkt1248 24297251 PMC3964941

[B42] Li X. Ao J. Wu J. (2017a). Systematic identification and comparison of expressed profiles of lncRNAs and circRNAs with associated co-expression and ceRNA networks in mouse germline stem cells. *Oncotarget* 8 26573–26590. 10.18632/oncotarget.15719 28404936 PMC5432280

[B43] Li X. Liu C.-X. Xue W. Zhang Y. Jiang S. Yin Q.-F. (2017b). Coordinated circRNA biogenesis and function with NF90/NF110 in viral infection. *Mol. Cell* 67 214–227. 10.1016/j.molcel.2017.05.023 28625552

[B44] Li Y. Li S. Li R. Xu J. Jin P. Chen L. (2017). Genome-wide miRNA screening reveals miR-310 family members negatively regulate the immune response in *Drosophila melanogaster* via co-targeting, *Drosomycin*. *Dev. Compar. Immunol.* 68 34–45. 10.1016/j.dci.2016.11.014 27871832

[B45] Li Z. Huang C. Bao C. Chen L. Lin M. Wang X. (2015). Exon-intron circular RNAs regulate transcription in the nucleus. *Nat. Struct. Mol. Biol.* 22 256–264. 10.1038/nsmb.2959 25664725

[B46] Liang S. Haitao L. Dechao B. Guoguang Z. Kuntao Y. Changhai Z. (2013). Utilizing sequence intrinsic composition to classify protein-coding and long non-coding transcripts. *Nucleic Acids Res.* 41:e166. 10.1093/nar/gkt646 23892401 PMC3783192

[B47] Ling L. Kokoza V. A. Zhang C. Aksoy E. Raikhel A. S. (2017). MicroRNA-277 targets insulin-like peptides 7 and 8 to control lipid metabolism and reproduction in *Aedes aegypti* mosquitoes. *Proc. Natl. Acad. Sci. U.S.A.* 114:E8017. 10.1073/pnas.1710970114 PMC5617303 28874536

[B48] Ling-Ling C. Li Y. (2015). Regulation of circRNA biogenesis. *RNA Biol.* 12 381–388. 10.1080/15476286.2015.1020271 25746834 PMC4615371

[B49] Long Y. Wang X. Youmans D. T. Cech T. R. (2017). How do lncRNAs regulate transcription? *Sci. Adv.* 3:eaao2110. 10.1126/sciadv.aao2110 28959731 PMC5617379

[B50] Lu Y. Wu K. Jiang Y. Xia B. Li P. Feng H. (2010). Mirid bug outbreaks in multiple crops correlated with wide-scale adoption of Bt cotton in China. *Science* 328 1151–1154. 10.1126/science.1187881 20466880

[B51] Luo D. Lai M. Xu C. Shi H. Liu X. (2018). Life history traits in a capital breeding pine caterpillar: effect of host species and needle age. *BMC Ecol.* 18:24 10.1186/s12898-018-0181-180 PMC6083516 30089520

[B52] Lv J. Cui W. Liu H. He H. Xiu Y. Guo J. (2013). Identification and characterization of long non-coding RNAs related to mouse embryonic brain development from available transcriptomic data. *PLoS One* 8:e71152 10.1371/journal.pone.00071152 PMC3743905 23967161

[B53] Maeda R. K. Sitnik J. L. Frei Y. Prince E. Gligorov D. Wolfner M. F. (2018). The lncRNA male-specific abdominal plays a critical role in *Drosophila* accessory gland development and male fertility. *PLoS Genet.* 14:e1007519. 10.1371/journal.pgen.1007519 30011265 PMC6067764

[B54] Marchese F. P. Raimondi I. Huarte M. (2017). The multidimensional mechanisms of long noncoding RNA function. *Genome Biol.* 18 206–206. 10.1186/s13059-017-1348-1342 29084573 PMC5663108

[B55] Mehta A. Baltimore D. (2016). MicroRNAs as regulatory elements in immune system logic. *Nat. Rev. Immunol.* 16:279. 10.1038/nri.2016.40 27121651

[B56] Mercer T. R. Mattick J. S. (2013). Structure and function of long noncoding RNAs in epigenetic regulation. *Nat. Struct. Mol. Biol.* 20 300–307. 10.1038/nsmb.2480 23463315

[B57] Meuti M. Bautista-Jimenez R. Reynolds J. (2018). MicroRNAs are likely part of the molecular toolkit regulating adult reproductive diapause in the mosquito, *Culex pipiens*. *PLoS One* 13:e0203015 10.1371/journal.pone.00203015 PMC6264513 30496183

[B58] Mowel W. K. Kotzin J. J. McCright S. J. Neal V. D. Henao-Mejia J. (2018). Control of immune cell homeostasis and function by lncRNAs. *Trends Immunol.* 39 55–69. 10.1016/j.it.2017.08.009 28919048 PMC5748345

[B59] Myers J. H. (1993). Population outbreaks in forest Lepidoptera. *Am. Sci.* 81 240–251.

[B60] Nan L. Michael L. Kajia C. Masashi A. Gert-Jan H. Kennerdell J. R. (2012). The microRNA miR-34 modulates ageing and neurodegeneration in *Drosophila*. *Nature* 482 519–523. 10.1038/nature10810 22343898 PMC3326599

[B61] Pener M. P. Simpson S. J. (2009). Locust phase polyphenism: an update. *Adv. Insect Physiol.* 36 1–72.

[B62] Pertea M. Kim D. Pertea G. M. Leek J. T. Salzberg S. L. (2016). Transcript-level expression analysis of RNA-seq experiments with HISAT, StringTie and Ballgown. *Nat. Protoc.* 11:1650. 10.1038/nprot.2016.095 27560171 PMC5032908

[B63] Priyamvada R. Ferdinando F. Robert S. Doron B. Puthanveettil S. V. Russo J. J. (2009). Characterization of small RNAs in *Aplysia* reveals a role for miR-124 in constraining synaptic plasticity through CREB. *Neuron* 63 803–817. 10.1016/j.neuron.2009.05.029 19778509 PMC2875683

[B64] Quezada García R. Seehausen M. L. Bauce É (2015). Adaptation of an outbreaking insect defoliator to chronic nutritional stress. *J. Evol. Biol.* 28 347–355. 10.1111/jeb.12571 25510541

[B65] Rehmsmeier M. Steffen P. Hochsmann M. Giegerich R. (2004). Fast and effective prediction of microRNA/target duplexes. *RNA* 10 1507–1517. 10.1261/rna.5248604 15383676 PMC1370637

[B66] Riffo-Campos ÁL. Riquelme I. Brebi-Mieville P. (2016). Tools for sequence-based miRNA target prediction: what to choose? *Intern. J. Mol. Sci.* 17:1987. 10.3390/ijms17121987 27941681 PMC5187787

[B67] Rinn J. L. Chang H. Y. (2012). Genome regulation by long noncoding RNAs. *Annu. Rev. Biochem.* 81 145–166. 10.1146/annurev-biochem-051410-092902 22663078 PMC3858397

[B68] Roy S. Saha T. T. Zou Z. Raikhel A. S. (2018). Regulatory pathways controlling female insect reproduction. *Annu. Rev. Entomol.* 63 489–511. 10.1146/annurev-ento-020117-043258 29058980

[B69] Salmena L. Poliseno L. Tay Y. Kats L. Pandolfi P. P. (2011). A ceRNA hypothesis: the rosetta stone of a hidden RNA language? *Cell* 146 353–358. 10.1016/j.cell.2011.07.014 21802130 PMC3235919

[B70] Stefan G. Juan Miguel G. G. Javier T. Williams T. D. Nagaraj S. H. María José N. (2008). High-throughput functional annotation and data mining with the Blast2GO suite. *Nucleic Acids Res.* 36 3420–3435. 10.1093/nar/gkn176 18445632 PMC2425479

[B71] Veran S. Simpson S. J. Sword G. A. Deveson E. Piry S. Hines J. E. (2015). Modeling spatiotemporal dynamics of outbreaking species: influence of environment and migration in a locust. *Ecology* 96 737–748. 10.1890/14-0183.1 26236870

[B72] Wang J. Ren Q. Hua L. Chen J. Zhang J. Bai H. (2019). Comprehensive analysis of differentially expressed mRNA, lncRNA and circRNA and their ceRNA networks in the longissimus dorsi muscle of two different pig breeds. *Intern. J. Mol. Sci.* 20:1107. 10.3390/ijms20051107 30836719 PMC6429497

[B73] Wang X. Fang X. Yang P. Jiang X. Jiang F. Zhao D. (2014). The locust genome provides insight into swarm formation and long-distance flight. *Nat. Commun.* 5:2957. 10.1038/ncomms3957 24423660 PMC3896762

[B74] Wang Y. Wang Q. Gao L. Zhu B. Luo Y. Deng Z. (2017). Integrative analysis of circRNAs acting as ceRNAs involved in ethylene pathway in tomato. *Physiol. Plant.* 161 311–321. 10.1111/ppl.12600 28664538

[B75] Wang Y. Yang P. Cui F. Kang L. (2013). Altered Immunity in Crowded Locust Reduced Fungal (*Metarhizium anisopliae*) Pathogenesis. *PLoS Pathog.* 9:e1003102 10.1371/journal.pone.01003102 PMC3542111 23326229

[B76] Wei Y. Chen S. Yang P. Ma Z. Kang L. (2009). Characterization and comparative profiling of the small RNA transcriptomes in two phases of locust. *Genome Biol.* 10:R6. 10.1186/gb-2009-10-1-r6 19146710 PMC2687794

[B77] Wen K. Yang L. Xiong T. Di C. Ma D. Wu M. (2016). Critical roles of long noncoding RNAs in *Drosophila* spermatogenesis. *Genome Res.* 26 1233–1244. 10.1101/gr.199547.115 27516619 PMC5052038

[B78] Westholm J. O. Miura P. Olson S. Shenker S. Joseph B. Sanfilippo P. (2014). Genome-wide analysis of *Drosophila* circular RNAs reveals their structural and sequence properties and age-dependent neural accumulation. *Cell Rep.* 9 1966–1980. 10.1016/j.celrep.2014.10.062 25544350 PMC4279448

[B79] Wilusz J. E. Sharp P. A. (2013). A circuitous route to noncoding RNA. *Science* 340 440–441. 10.1126/science.1238522 23620042 PMC4063205

[B80] Wu Y. Cheng T. Liu C. Liu D. Zhang Q. Long R. (2016). Systematic identification and characterization of long non-coding RNAs in the silkworm, *Bombyx mori*. *PLoS One* 11:e0147147. 10.1371/journal.pone.0147147 26771876 PMC4714849

[B81] Xiao G. (1992). *Forest Insects of China.* Beijing: China Forestry Publishing House.

[B82] Xie C. Mao X. Huang J. Ding Y. Wu J. Dong S. (2011). KOBAS 2.0: a web server for annotation and identification of enriched pathways and diseases. *Nucleic Acids Res.* 39 316–322. 10.1093/nar/gkr483 PMC3125809 21715386

[B83] Xiu Y. Jiang G. Zhou S. Diao J. Liu H. Su B. (2019). Identification of potential immune-related circRNA-miRNA-mRNA regulatory network in intestine of *Paralichthys olivaceus* during *Edwardsiella tarda* Infection. *Front. Genet.* 10:731. 10.3389/fgene.2019.00731 31475036 PMC6702444

[B84] Yang C.-H. Yang P.-C. Li J. Yang F. Zhang A.-B. (2016). Transcriptome characterization of *Dendrolimus punctatus* and expression profiles at different developmental stages. *PLoS One* 11:e0161667. 10.1371/journal.pone.0161667 27560151 PMC4999207

[B85] Yang C.-H. Yang P.-C. Zhang S.-F. Shi Z.-Y. Kang L. Zhang A.-B. (2017). Identification, expression pattern, and feature analysis of cuticular protein genes in the pine moth *Dendrolimus punctatus* (Lepidoptera: Lasiocampidae). *Insect Biochem. Mol. Biol.* 83(Suppl. C), 94–106. 10.1016/j.ibmb.2017.03.003 28284855

[B86] Yang Y. Fan X. Mao M. Song X. Wu P. Zhang Y. (2017). Extensive translation of circular RNAs driven by N6-methyladenosine. *Cell Res.* 27:626. 10.1038/cr.2017.31 28281539 PMC5520850

[B87] You X. Vlatkovic I. Babic A. Will T. Epstein I. Tushev G. (2015). Neural circular RNAs are derived from synaptic genes and regulated by development and plasticity. *Nat. Neurosci.* 18 603–610. 10.1038/nn.3975 25714049 PMC4376664

[B88] Yvonne T. John R. Pier Paolo P. (2014). The multilayered complexity of ceRNA crosstalk and competition. *Nature* 505 344–352. 10.1038/nature12986 24429633 PMC4113481

[B89] Zhang S. Kong X. Ze S. Wang H. Lin A. Liu F. (2016). Discrimination of cis-trans sex pheromone components in two sympatric Lepidopteran species. *Insect Biochem. Mol. Biol.* 73 47–54. 10.1016/j.ibmb.2016.04.004 27107681

[B90] Zhang S. Shen S. Peng J. Zhou X. Kong X. Ren P. (2020). Chromosome-level genome assembly of an important pine defoliator, *Dendrolimus punctatus* (Lepidoptera; Lasiocampidae). *Mol. Ecol. Resour.* Accepted. 10.1111/1755-0998.13169 32306534

[B91] Zhang S.-F. Zhang Z. Kong X.-B. Wang H.-B. Liu F. (2018). Dynamic changes in chemosensory gene expression during the *Dendrolimus punctatus* mating process. *Front. Physiol.* 8:1127. 10.3389/fphys.2017.01127 29375398 PMC5767605

[B92] Zhang Y. Zhang X. O. Chen T. Xiang J. F. Yin Q. F. Xing Y. H. (2013). Circular intronic long noncoding RNAs. *Mol. Cell* 51 792–806. 10.1016/j.molcel.2013.08.017 24035497

[B93] Zhang Z. Li D. Cha G. (2002). Time series analysis and complex dynamics of mason pine caterpillar, *Dendrolimus punctatus* walker (Lepidoptera: Lasiocampidae). *Acta Ecol. Sin.* 22 1061–1067.

[B94] Zhang Z. Tang J. He X. Zhu M. Gan S. Guo X. (2019). Comparative transcriptomics identify key hypothalamic circular RNAs that participate in sheep (*Ovis aries*) reproduction. *Animals* 9:557. 10.3390/ani9080557 31416269 PMC6721059

