# Supplementary figures and images for: Coding and Non-coding RNAs: Molecular Basis of Forest-Insect Outbreaks
Source: Front Cell Dev Biol. 2020 Jun 11;8:369. doi: 10.3389/fcell.2020.00369 (PMC7300193; doi:10.3389/fcell.2020.00369)

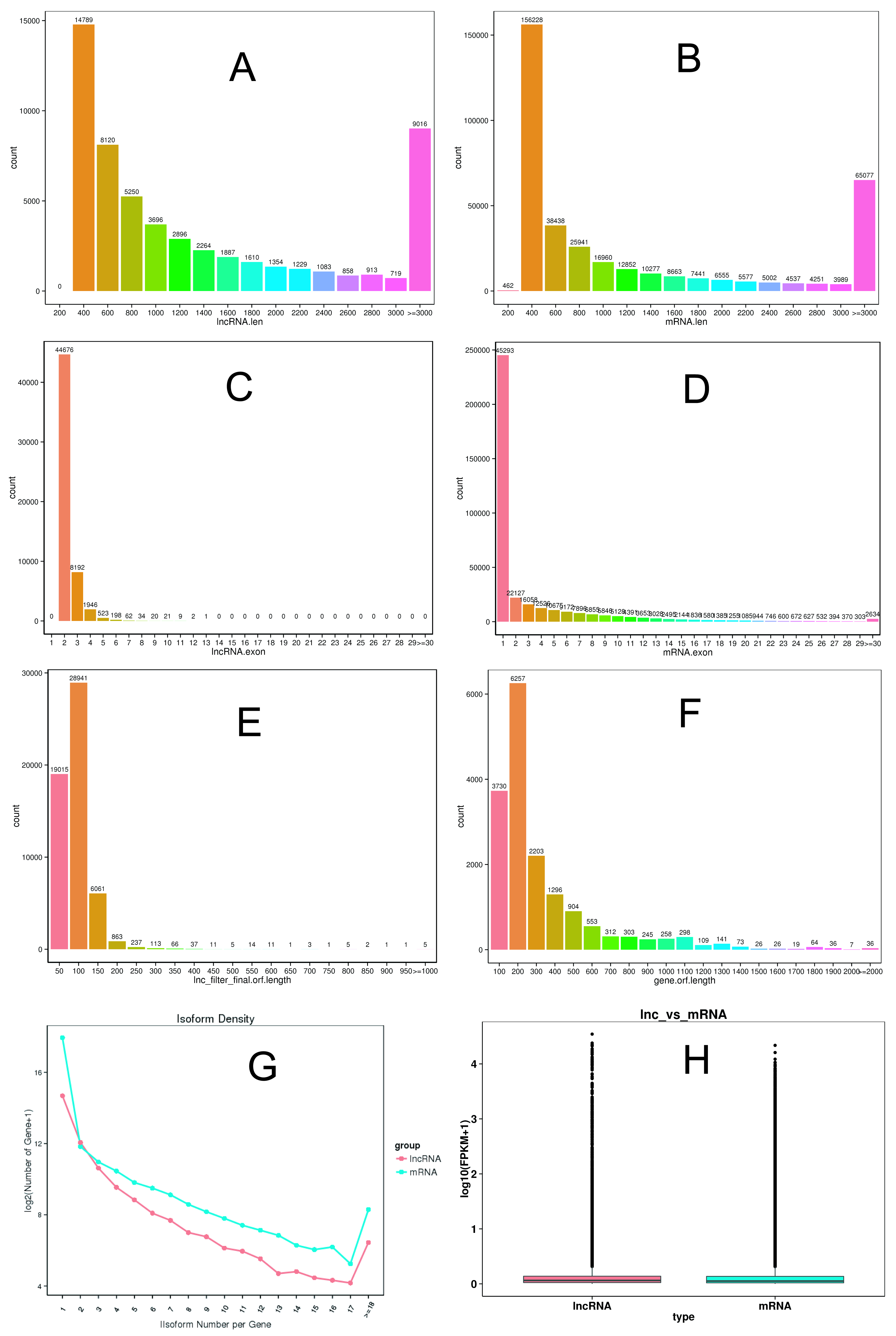

Supplement: FIGURE S1 — Structural features and expression comparisons of lncRNAs and mRNAs in Dendrolimus punctatus. (A) Lengths of lncRNAs; (B) lengths of mRNAs; (C) exon numbers of lncRNAs; (D) exon numbers of mRNAs; (E) ORF lengths of lncRNAs; (F) ORF lengths of mRNAs; (G) the alternatively spliced isoforms of lncRNAs and mRNAs; (H) the expression levels of lncRNAs and mRNAs. [file Image_1.JPEG]

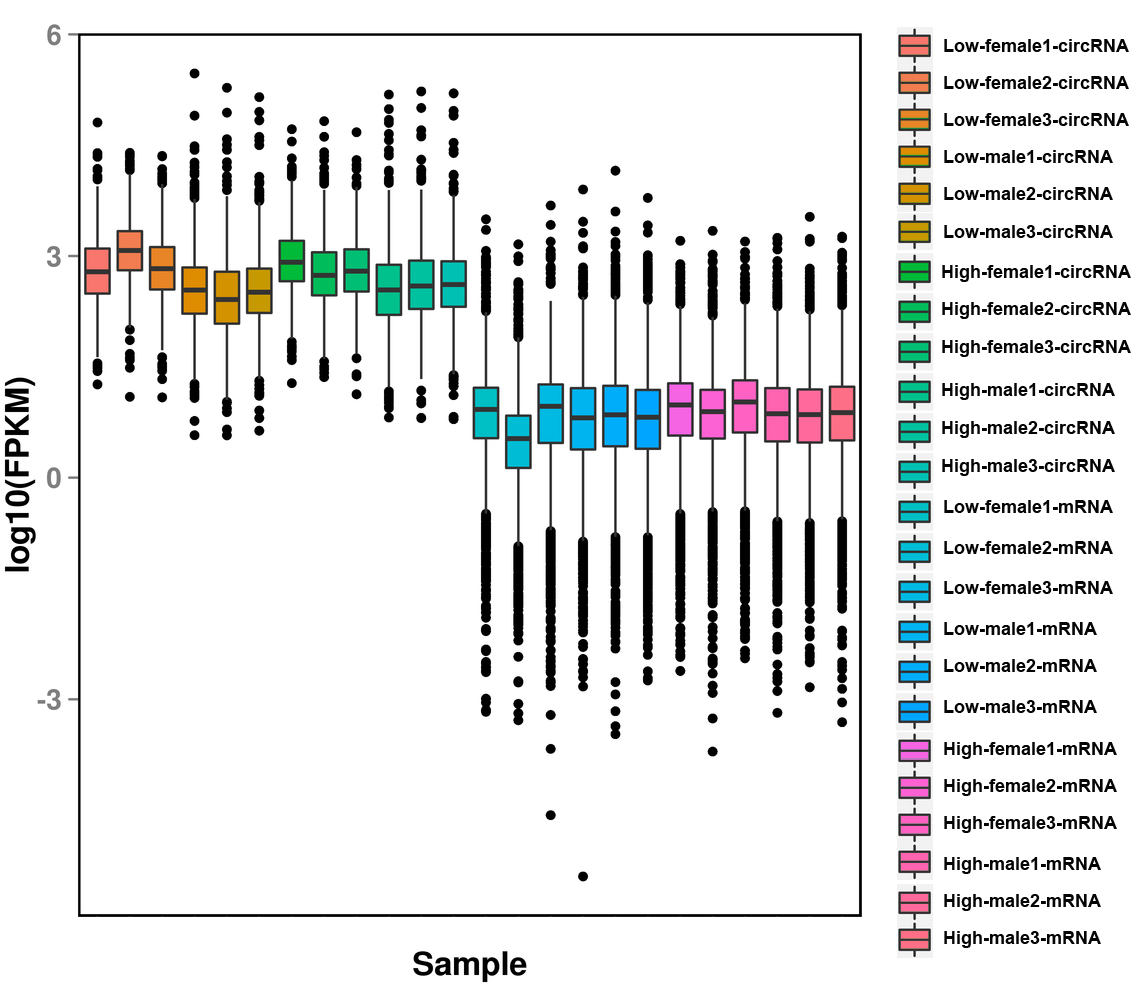

Supplement: FIGURE S2 — FPKM comparisons for the mRNAs and circRNAs. [file Image_2.JPEG]

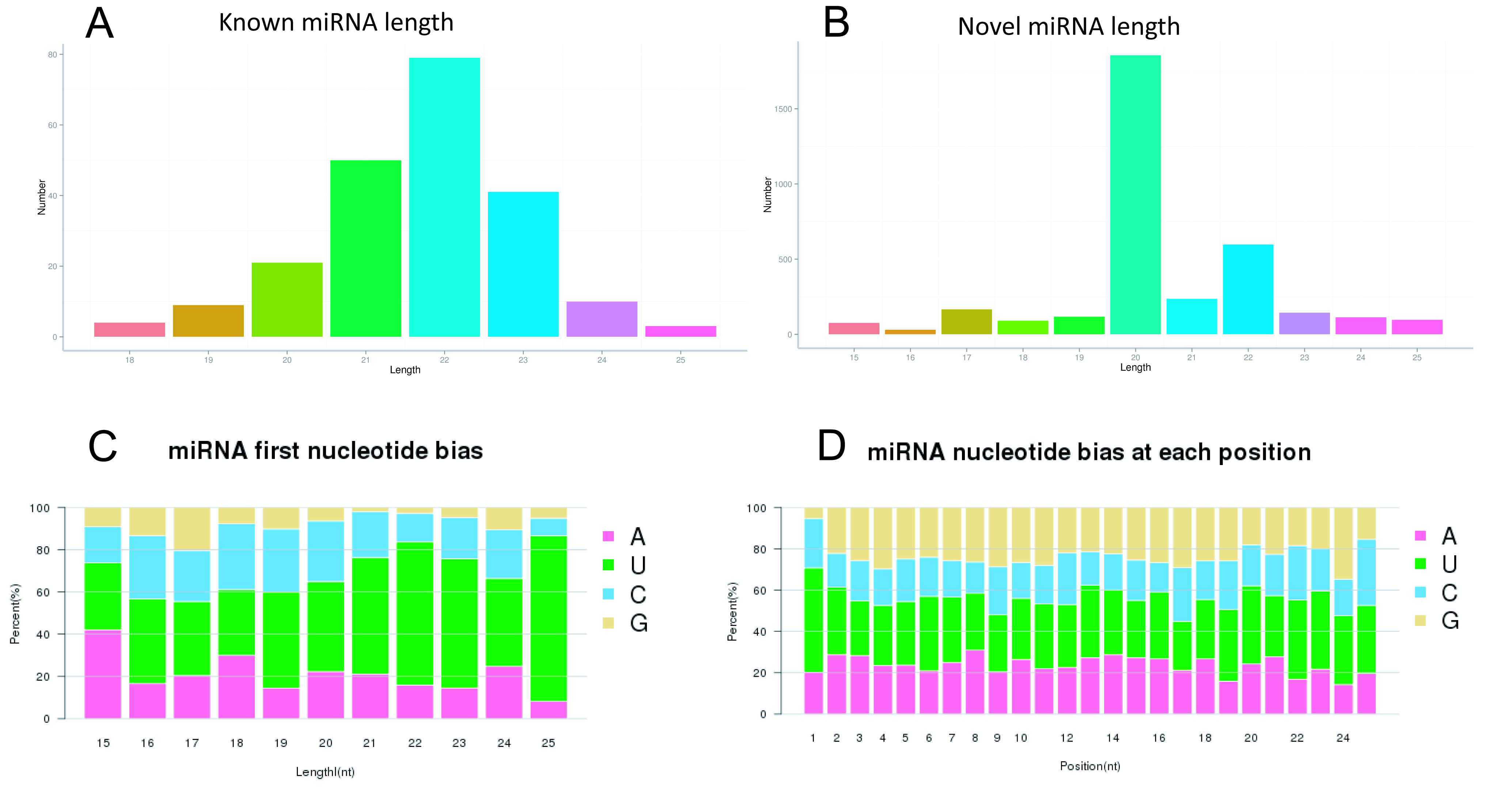

Supplement: FIGURE S3 — Characterization of miRNAs in Dendrolimus punctatus. (A,B) Length distributions of known (A) and novel (B) miRNAs identified in this study; (C) first nucleotide bias of miRNAs; (D) nucleotide bias analysis at each miRNA position. [file Image_3.JPEG]

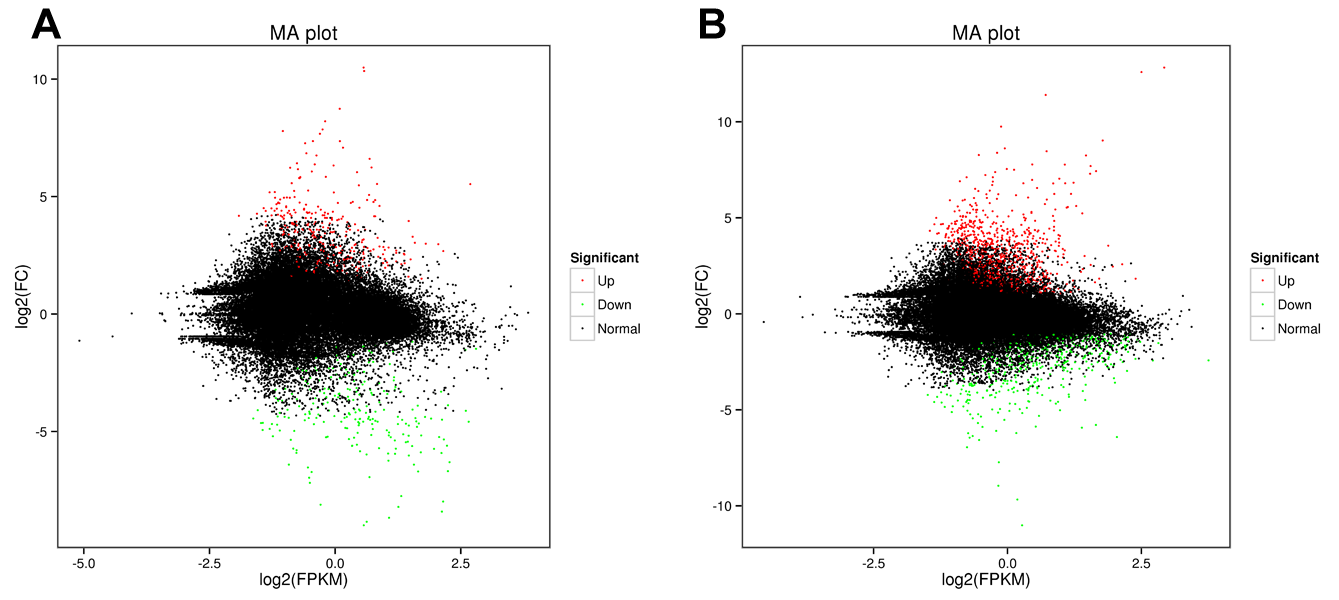

Supplement: FIGURE S4 — MA plot of the differences between low- and high-density Dendrolimus punctatus in females (A) and males (B). [file Image_4.TIF]

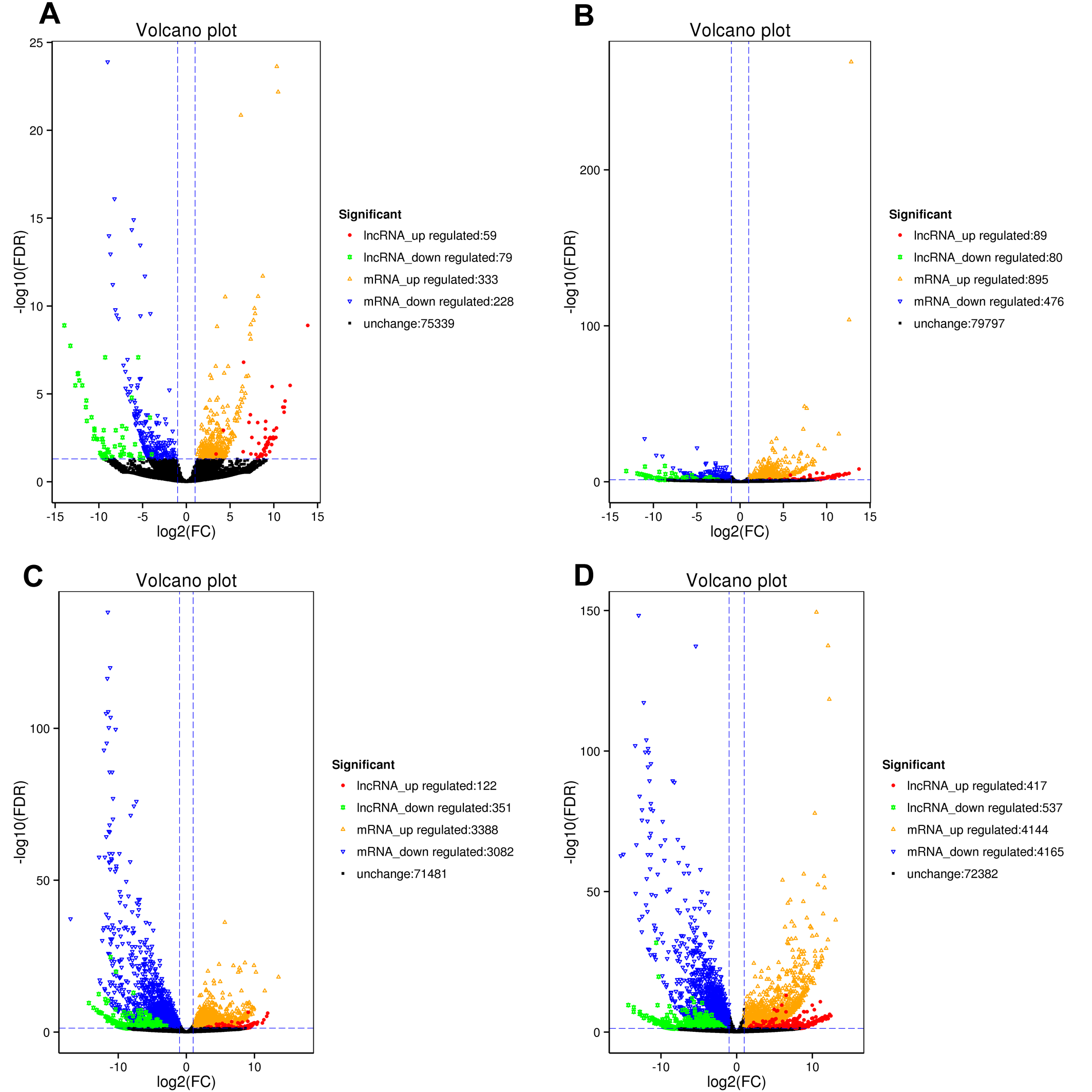

Supplement: FIGURE S5 — Volcano plot of mRNA and lncRNA-targeted DEGs in Dendrolimus punctatus between low- vs. high-density population in females (A), males (B), and between sexes in low- (C) and high- (D) density populations. [file Image_5.TIF]

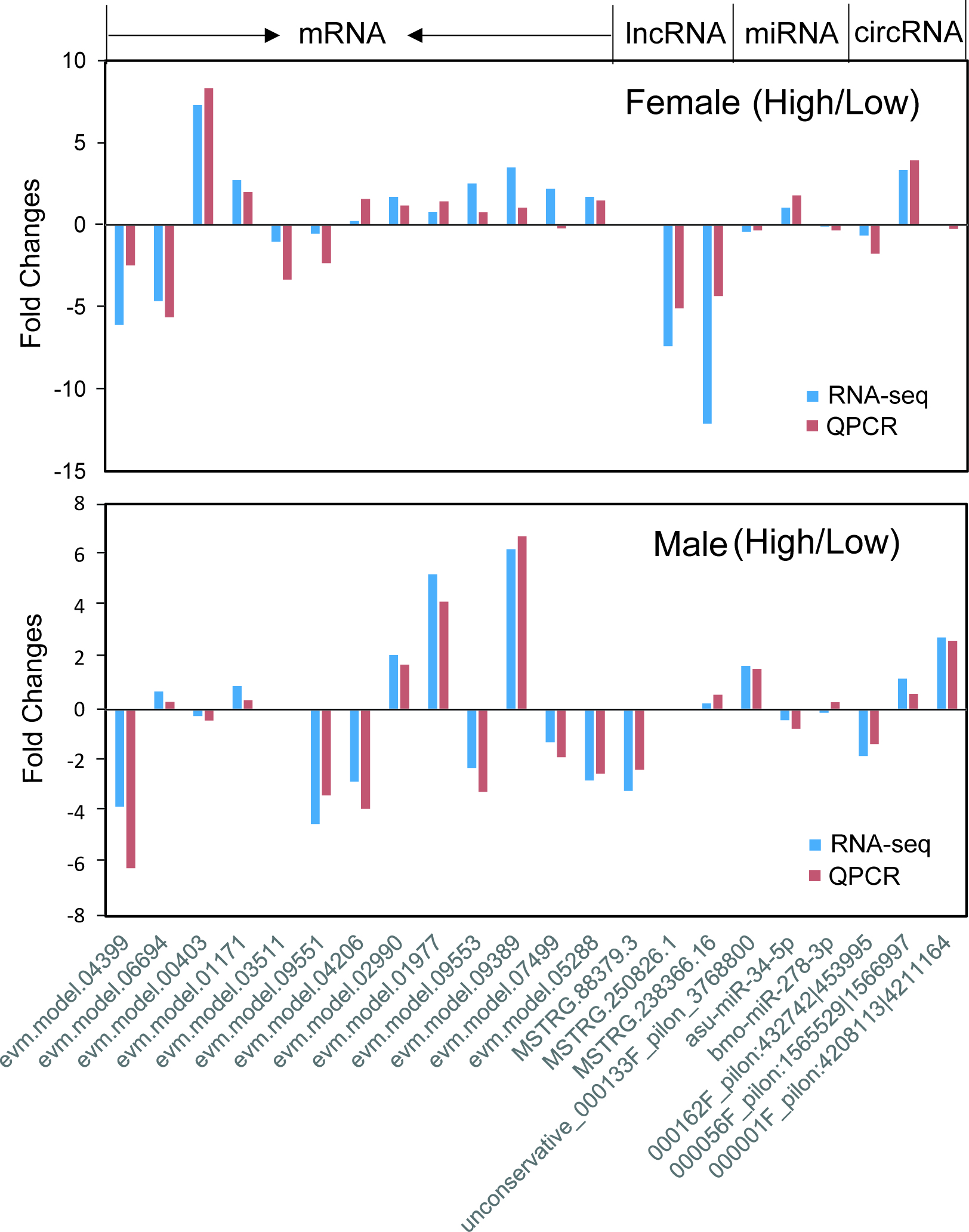

Supplement: FIGURE S6 — Real-time PCR validation of the RNA-Seq data. The x-axis shows the RNA names, and the y-axis is their log2 (fold change) based on the ratio of high-density and low-density insects. [file Image_6.JPEG]

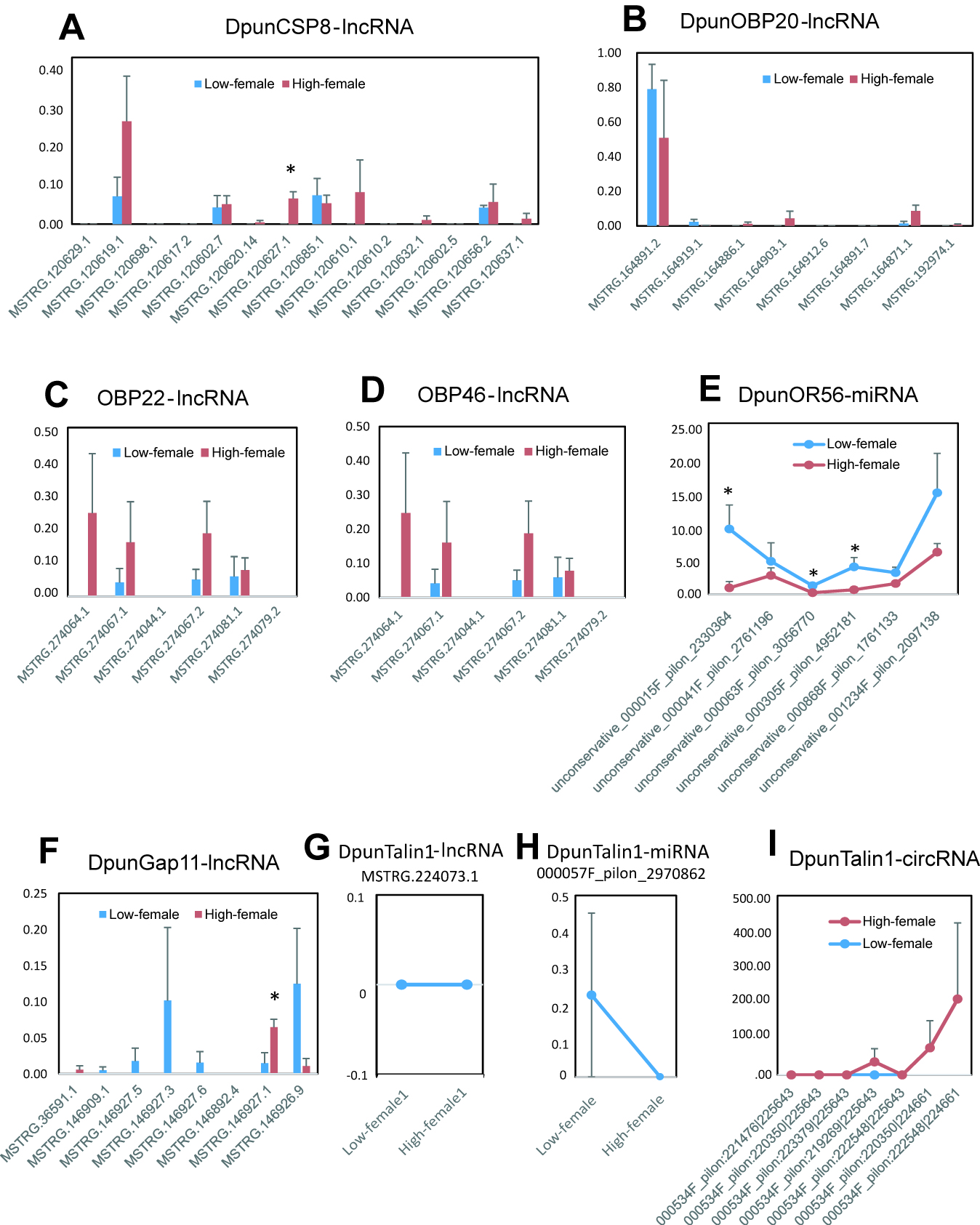

Supplement: FIGURE S7 — All non-coding RNA regulators of differently expressed chemosensory and immune genes between low- vs. high-density females of Dendrolimus punctatus. (A–D) lncRNA regulators of DpunCSP8, DpunOBP20, DpunOBP22, DpunOBP46; (E) miRNA regulators of DpunOR56; (F) lncRNA regulators of DpunGap11; (G–I) lncRNA, miRNA, and circRNA regulators of DpunTalin1. [file Image_7.JPEG]

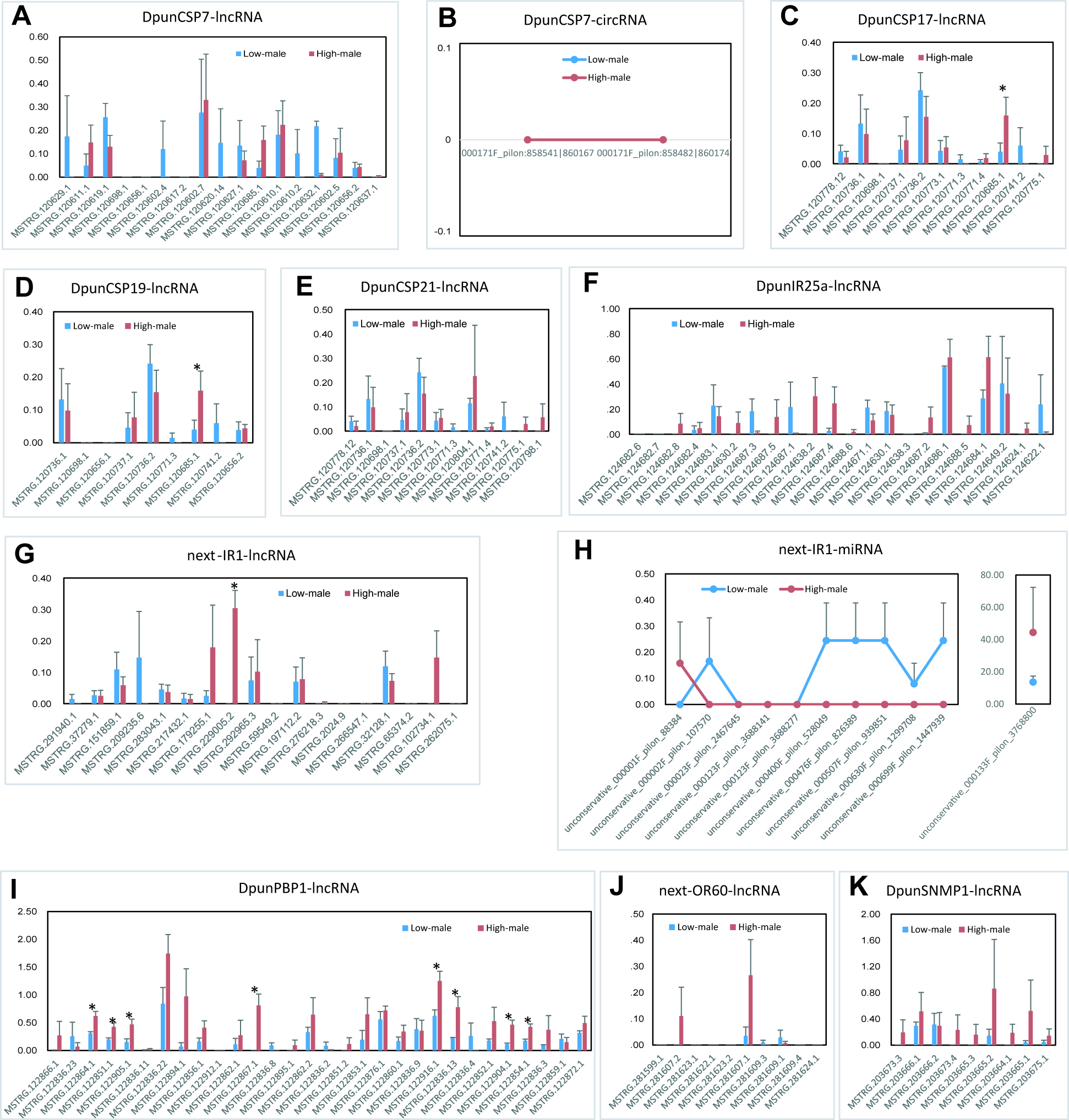

Supplement: FIGURE S8 — All non-coding RNA regulators of differently expressed chemosensory genes between low- vs. high-density males of Dendrolimus punctatus. (A,B) lncRNA and circRNA regulators of DpunCSP7; (C–F) lncRNA regulators of DpunCSP17, DpunCSP19, DpunCSP21, and DpunIR25a; (G,H) lncRNA and miRNA regulators of DpunNext-IR1; (I–K) lncRNA regulators of DpunPBP1, DpunNext-OR60, and DpunSNPM1. [file Image_8.JPEG]

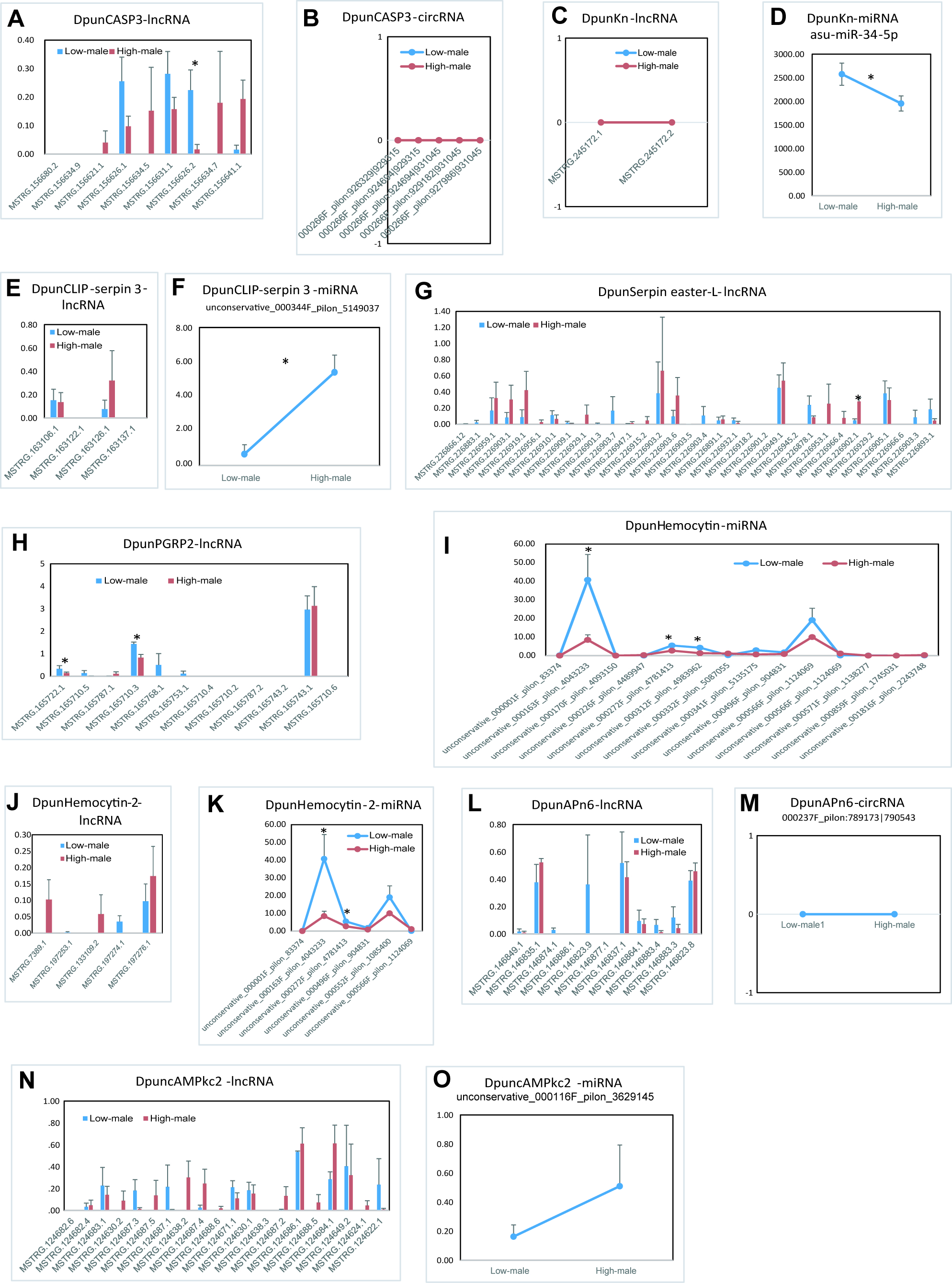

Supplement: FIGURE S9 — All non-coding RNA regulators of differently expressed immune genes between low- vs. high-density males of Dendrolimus punctatus. (A,B) lncRNA and circRNA regulators of DpunCASP3; (C,D) lncRNA and miRNA regulators of DpunKn; (E,F) lncRNA and miRNA regulators of DpunCLIP-serpin 3; (G,H) lncRNA regulators of DpunSerpin easter-L and DpunPGRP2; (I) miRNA regulators of DpunHemocytin; (J,K) lncRNA and miRNA regulators of DpunHemocytin-2; (L,M) lncRNA and circRNA regulators of DpunAPn6; (N,O) lncRNA and miRNA regulators of DpuncAMPkc2. [file Image_9.JPEG]
