# Supplementary material for: Coding and Non-coding RNAs: Molecular Basis of Forest-Insect Outbreaks
Source: Front Cell Dev Biol. 2020 Jun 11;8:369. doi: 10.3389/fcell.2020.00369 (PMC7300193; doi:10.3389/fcell.2020.00369)
Supplement: TABLE S1 — Primers used for the gene expression validation with Real-time PCR. [file Table_1.DOCX]

**Table S1.** Primers used for the gene expression validation with Real-time PCR.

| **RNAs** | **Primer (5'—3')** | |
| --- | --- | --- |
| evm.model.04399 | forward | CGTGTTACGGGGTTCT |
|  | reverse | CTAATTTGATCGTTGCTCT |
| evm.model.06694 | forward | GACTCTAATGTAAACCCACAA |
|  | reverse | ATGCCAAAGTATGCTGAAC |
| evm.model.00403 | forward | TGGCAACAACACTGAC |
|  | reverse | TTTGATAACCTAACCCT |
| evm.model.01171 | forward | AGGATGTAGCCTTAGCA |
|  | reverse | GATTGCGTGTTTTGG |
| evm.model.03511 | forward | GATAGTGATGCCTTGGG |
|  | reverse | GTAGATTTCGGATTTGTTG |
| evm.model.09551 | forward | ATGAGGGATTGGTTGTTAT |
|  | reverse | AATTCTTGCCGTCTTTAGT |
| evm.model.04206 | forward | AACAGGAAATCGGAAAG |
|  | reverse | GTAGTAACATCAGCCAGAA |
| evm.model.02990 | forward | AGCCGATCATCACCCA |
|  | reverse | CGCCAGCTTGTACTGC |
| evm.model.01977 | forward | CGCTCAAGACGACAGA |
|  | reverse | GAGGCGACAGGAAATAC |
| evm.model.09553 | forward | TCTGGACGCCTATTGAA |
|  | reverse | CGCATCGGGTAGAGTTT |
| evm.model.09389 | forward | TTCGTAAGTTATTGGCGTGAG |
|  | reverse | CAGGCGTGGATGGTTTC |
| evm.model.07499 | forward | GAGGCAAACTCATGGACG |
|  | reverse | TTCAACAGTGGAGCATAAGAC |
| evm.model.05288 | forward | CACTCGCTGCCGTTCTAC |
|  | reverse | CTTCGCAATGTTCTACTGTCTT |
| MSTRG.88379.3 | forward | CCGTAATCACCCTCG |
|  | reverse | AACATCAATCTACCTCGT |
| MSTRG.250826.1 | forward | TGCCAACAGCGTCAA |
|  | reverse | GTGGGAAGAGTAGGAACAT |
| MSTRG.238366.16 | forward | GCCTTATTAGATTGAACG |
|  | reverse | ACAAACGTCGGAACA |
| unconservative_000133F  _pilon_3768800 | forward | CGTGCCGTGTCTGTACATTGAAGC |
|  | reverse | ATCCAGTGCAGGGTCCGAGG |
| asu-miR-34-5p | forward | TGGCAGTGTGGTTAGCTGGTTGT |
|  | reverse | ATCCAGTGCAGGGTCCGAGG |
| bmo-miR-278-3p | forward | TCGGTGGGATCTTCGTCCGTTT |
|  | reverse | ATCCAGTGCAGGGTCCGAGG |
| 000162F_pilon:  432742\|453995 | forward | CCCGAATGGTCTACGCCTACTA |
|  | reverse | TTTTACAGCGGAACTC |
| 000056F_pilon:  1565529\|1566997 | forward | GCAAGAGTGGAGATGAGAA |
|  | reverse | AGCCCTGTGGAATAGTTT |
| 000001F_pilon:  4208113\|4211164 | forward | GGATTGCCTTGGCTGAT |
|  | reverse | CAACTCGGGTGCTGACG |
| U6 | forward | GGCGTGACAGGTACATATACTAA |
|  | reverse | ATCCAGTGCAGGGTCCGAGG |
| Dpunbeta-Actin | forward | GCGATCTTACCGACTACCTCA |
|  | reverse | TCTGGGCAACGGAACCT |

Table S2. Summary of the sequenced data for *D. punctatus*.

| **Samples** | **ReadSum** | **BaseSum** | **GC(%)** | **N(%)** | **Q30(%)** |
| --- | --- | --- | --- | --- | --- |
| Low-♀-1 | 63967956 | 18951255330 | 45.28 | 0.00 | 94.21 |
| Low-♀-2 | 55998446 | 16652891700 | 50.53 | 0.00 | 92.99 |
| Low-♀-3 | 65842199 | 19530161582 | 45.97 | 0.00 | 93.69 |
| Low-♂-1 | 55691609 | 16592648140 | 42.91 | 0.00 | 94.03 |
| Low-♂-2 | 61523760 | 18362298954 | 41.69 | 0.00 | 94.35 |
| Low-♂-3 | 58685499 | 17536536994 | 42.87 | 0.00 | 94.29 |
| High-♀-1 | 55222763 | 16448104384 | 43.79 | 0.00 | 93.58 |
| High-♀-2 | 54582582 | 16282748320 | 44.53 | 0.00 | 94.30 |
| High-♀-3 | 57876820 | 17236335390 | 42.77 | 0.00 | 94.39 |
| High-♂-1 | 56447639 | 16849622552 | 41.85 | 0.00 | 93.71 |
| High-♂-2 | 55795128 | 16572260964 | 43.56 | 0.00 | 94.46 |
| High-♂-3 | 60897412 | 18228361806 | 42.67 | 0.00 | 93.54 |

Low: samples from low population density; High: samples from high population density; ReadSum: total number of pair-end Reads from Clean Data; BaseSum：total base from Clean Data; GC(%)：GC amount from; N(%): percentage of unresolved bases in clean data; Q30(%): percentage of bases whose quality is greater than or equal to Q30 in clean data。

Table S3. Sequence alignment results for the comparison of sequencing data to selected reference genomes.

| **Samples** | **Total Reads** | **Mapped Reads** | **Uniq Mapped Reads** | **Multiple Mapped Reads** | **Reads Map to '+'** | **Reads Map to '-'** |
| --- | --- | --- | --- | --- | --- | --- |
| Low-♀-1 | 127935912 | 112199115(87.70%) | 89915160(70.28%) | 22283955(17.42%) | 50212344(39.25%) | 50170877(39.22%) |
| Low-♀-2 | 111996892 | 102018899(91.09%) | 40759488(36.39%) | 61259411(54.70%) | 29147933(26.03%) | 29229303(26.10%) |
| Low-♀-3 | 131684398 | 116994675(88.84%) | 90475156(68.71%) | 26519519(20.14%) | 51824118(39.35%) | 51482423(39.10%) |
| Low-♂-1 | 111383218 | 84238384(75.63%) | 71136011(63.87%) | 13102373(11.76%) | 38043087(34.16%) | 38010263(34.13%) |
| Low-♂-2 | 123047520 | 104085078(84.59%) | 88063254(71.57%) | 16021824(13.02%) | 47199127(38.36%) | 47145102(38.31%) |
| Low-♂-3 | 117370998 | 80608975(68.68%) | 68291731(58.18%) | 12317244(10.49%) | 36789120(31.34%) | 36729458(31.29%) |
| High-♀-1 | 110445526 | 95173196(86.17%) | 77348448(70.03%) | 17824748(16.14%) | 42559403(38.53%) | 42553144(38.53%) |
| High-♀-2 | 109165164 | 95920934(87.87%) | 76384459(69.97%) | 19536475(17.90%) | 42457007(38.89%) | 42439170(38.88%) |
| High-♀-3 | 115753640 | 100807566(87.09%) | 82737678(71.48%) | 18069888(15.61%) | 44860757(38.76%) | 44841096(38.74%) |
| High-♂-1 | 112895278 | 91696389(81.22%) | 77643418(68.77%) | 14052971(12.45%) | 41741912(36.97%) | 41757692(36.99%) |
| High-♂-2 | 111590256 | 87846898(78.72%) | 69295729(62.10%) | 18551169(16.62%) | 37657737(33.75%) | 37653292(33.74%) |
| High-♂-3 | 121794824 | 96386790(79.14%) | 80297012(65.93%) | 16089778(13.21%) | 43224089(35.49%) | 43172996(35.45%) |

**Low**: samples from low population density; **High**: samples from high population density; **Total Reads**: total number of Clean Reads (single end); **Mapped Reads**: numbers and percentages of the clean reads mapped to the genome sequences; **Uniq Mapped Reads**: numbers and percentages of the clean reads mapped to an unique locate on the genome sequences; **Multiple Mapped Reads**: numbers and percentages of the clean reads mapped to multiple locates on the genome sequences; **Reads Map to '+'**: numbers and percentages of the clean reads mapped to “+” strand of the genome; **Reads Map to '-'**: numbers and percentages of the clean reads mapped to “-” strand of the genome.

Table S4. Sequence alignment results for the comparison of sequenced miRNAs to selected reference genomes.

| **Samples** | **Total_Reads** | **Mapped_Reads** | **Mapped_reads(+)** | **Mapped_reads(-)** |
| --- | --- | --- | --- | --- |
| Low-♀-1 | 12651048 | 8505310 | 6102757 | 2402553 |
| Low-♀-2 | 13995200 | 8612240 | 6377133 | 2235107 |
| Low-♀-3 | 15399503 | 9955189 | 8033664 | 1921525 |
| Low-♂-1 | 15038250 | 10273584 | 5532217 | 4741367 |
| Low-♂-2 | 14621717 | 9973089 | 6256547 | 3716542 |
| Low-♂-3 | 9262937 | 4836426 | 3354860 | 1481566 |
| High-♀-1 | 14930877 | 9148909 | 6838439 | 2310470 |
| High-♀-2 | 13685850 | 8445745 | 5950190 | 2495555 |
| High-♀-3 | 14841510 | 8566943 | 6296345 | 2270598 |
| High-♂-1 | 15461419 | 8437309 | 5764929 | 2672380 |
| High-♂-2 | 11220881 | 6655506 | 4309447 | 2346059 |
| High-♂-3 | 12860597 | 6382798 | 4407461 | 1975337 |

**Low**: samples from low population density; **High**: samples from high population density; **Total Reads**: total number of Clean Reads; **Mapped Reads**: numbers of the clean reads mapped to the genome sequences; **Reads Map to '+'**: numbers of the clean reads mapped to “+” strand of the genome; **Reads Map to '-'**: numbers of the clean reads mapped to “-” strand of the genome.

Table S5. circRNA, lncRNA, and mRNA gene numbers that correlated to miRNA.

| **Type** | **Known** | **Novel** | **All** |
| --- | --- | --- | --- |
| miRNA | 217 | 3521 | 3738 |
| circRNA | 84 | 2690 | 2774 |
| lncRNA | 126 | 2940 | 3066 |
| mRNA | 153 | 2990 | 3143 |
